# Molecular Interactions of Bioactive Peptides with Proteins and DNA

**DOI:** 10.3390/pharmaceutics18101311

**Published:** 2026-10-15

**Authors:** Aleksandra Kotynia, Edward Krzyżak, Żaneta Czyżnikowska, Aleksandra Marciniak

**Affiliations:** Department of Basic Chemical Sciences, Faculty of Pharmacy, Wroclaw Medical University, Borowska 211a, 50-556 Wrocław, Poland; edward.krzyzak@umw.edu.pl (E.K.); zaneta.czyznikowska@umw.edu.pl (Ż.C.); aleksandra.marciniak@umw.edu.pl (A.M.)

**Keywords:** snail peptides, serum albumin, acid glycoprotein, human immunoglobulin G, lysozyme, spectroscopic analyses, molecular docking

## Abstract

**Background:** This study investigates the molecular recognition of two biologically active peptides (P1 and P2) by key biomacromolecular targets. The studied peptides were previously identified in Babylon snail hydrolysates and have been shown to exhibit antioxidant and cytotoxic activities against Caco-2 colorectal cancer cells. Given their reported biological potential, we decided to investigate both compounds from a different perspective. Their interactions with human serum albumin (HSA), alpha-1-acid glycoprotein (AAG), lysozyme (LYS), immunoglobulin G (IgG), and DNA were evaluated. **Methods, Results and Conclusions:** Spectroscopic analyses revealed concentration-dependent binding of the studied peptides to plasma proteins under investigations. Notably, peptide binding did not substantially alter the secondary structure of the target proteins, indicating that complex formation occurs without significant structural perturbation. Molecular docking further supported the formation of peptide–protein complexes, highlighting the contribution of hydrogen bonding and hydrophobic contacts to molecular recognition. The combined spectroscopic and docking results were consistent with a predominantly non-intercalative, possibly minor-groove-associated interaction with ctDNA. Taken together, these findings provide a comparative physicochemical characterization of the interactions of P1 and P2 with selected proteins and ctDNA. These findings provide insight into the molecular recognition properties of P1 and P2 and support further investigation of their biological relevance.

## 1. Introduction

Cancer is a heterogeneous and dynamic disease, requiring drugs that interact with a variety of molecular targets and signaling pathways. Furthermore, the discovery of new compounds with tumor activity enables the development of innovative therapeutic strategies, including targeted and combination therapies. Many currently used drugs demonstrate limited efficacy and high toxicity to healthy tissues. The development of drug resistance in cancer cells is also a common problem, significantly reducing the effectiveness of therapy. Research on peptide compounds with anticancer activity is an important and rapidly developing area of modern oncology [1,2]. Peptides are characterized by high selectivity, which can lead to reduced toxicity to healthy cells. Small peptides exhibit new mechanisms of action, distinct from classic chemotherapeutics, offering the potential to overcome drug resistance [1].

The binding of drugs to plasma proteins is critical to their pharmacokinetics and pharmacodynamics. Determining the extent of this binding affects the efficacy, dosage, and safety of therapy. Substances bound to proteins cannot penetrate biological membranes or act on receptors, but they form a reservoir from which the drug is released gradually. The active fraction is the form not bound to proteins, which is then available to receptors and determines the pharmacological effect [3,4,5]. Compounds that bind strongly to proteins can significantly influence the pharmacokinetic behavior of drugs by affecting their free fraction, distribution, clearance, and consequently their apparent half-life. This is particularly relevant for several anticancer agents, including the alkylating drug melphalan and anthracyclines such as doxorubicin and daunorubicin. [6,7]. Compounds with low affinity for proteins act more quickly but for a shorter time [4,8]. Therefore, understanding the mechanism of protein interaction and considering the degree of protein binding are crucial for ensuring therapeutic efficacy.

The main cellular target of cytotoxicity is DNA. It is thought that the interaction between small molecules and DNA can lead to the blockage of cancer cell division, resulting in the arrest of cell proliferation and death. This strategy is the basis of many cancer treatments that aim to stop the uncontrolled growth of cancer cells. Small molecules can interact with DNA in various ways, which can lead to structural changes in DNA that interfere with its normal function. The binding of compounds to DNA can be covalent, such as that of cisplatin, or non-covalent, which is more common [9,10]. Non-covalent binding to DNA can be divided into several models. Small molecules can intercalate between DNA bases, which can distort the double helix of DNA, making it difficult for cells to replicate or repair DNA. This way of interaction is exhibited by doxorubicin, actinomycin D, and bleomycin [11]. Some compounds bind in the minor or major groove, which is usually a weaker binding mode than intercalation. Others can only interact at the surface by electrostatic forces. However, some structures are characterized by a mixed binding mechanism [10]. The thermodynamic parameters of DNA–drug complex formation provide valuable insights into the binding affinity, driving forces, and molecular determinants underlying the interactions of anticancer agents with DNA. These interactions may in turn influence the biological and therapeutic properties of the agents. [12,13,14,15]. The thermodynamics of molecular interactions of ligands with DNA is essential for understanding the mechanism of the medical and biological action of existing drugs and for developing new, more effective therapeutics.

In this work, we aimed to characterize two peptides with ctDNA and selected plasma proteins. These peptides consist of the following sequences: HTYHEVTKH (P1) and WPVLAYHF (P2), and were identified in fractions obtained from the protein hydrolysates of the *Babylonia areolata* snail. The two compounds mentioned have been shown to neutralize free radicals, thereby reducing DNA damage and oxidative stress, factors that promote cancer development. Additionally, they exhibit a cytotoxic effect on colon cancer cells (Caco-2) [16]. Based on these preliminary research results, it can be assumed that these compounds may have potential biological activity. However, further research in this area is required. For this reason, we decided to investigate the peptides from a completely different perspective. We wanted to examine their affinity for biologically significant biomacromolecules: plasma proteins (HSA, AAG, IgG and LYS) and deoxyribonucleic acid.

In order to elucidate the molecular basis of ligand recognition by biological macromolecules, a multiscale analytical framework is required. This framework must be capable of resolving both the thermodynamic and structural determinants of complex formation. In contemporary biochemistry and biophysics, spectroscopic techniques are increasingly integrated with computational modeling, as each methodology probes distinct physicochemical aspects of interactions. Each methodology interrogates a distinct physicochemical dimension of the interaction, and their convergence enables a comprehensive description of the molecular recognition process. The combination of spectroscopic techniques, including UV–visible absorption, fluorescence, and circular dichroism (CD) spectroscopy, collectively constitutes a powerful experimental platform for the analysis of ligand–biomacromolecule interactions. The application of these complementary techniques provides insights into binding equilibria, facilitates the characterization of quenching mechanisms, enables the quantification of changes in affinity, and captures ligand-induced perturbations in the secondary and tertiary organization of proteins or the conformational architecture of nucleic acids [17,18,19]. At the molecular level, these experimental observations are increasingly complemented by molecular docking, which predicts energetically favorable recognition modes and plausible binding regions [20,21]. Collectively, these approaches furnish insight into the intricate network of non-covalent interactions that collectively define binding specificity, affinity, and complex stability. Consequently, the prevailing paradigm for investigating ligand–biomacromolecule recognition is now the convergence of advanced multispectroscopy analyses with molecular modeling. This multidisciplinary strategy establishes a complementary experimental–computational framework, thereby enabling a comprehensive interpretation of molecular recognition and facilitating an understanding of the physicochemical principles that govern biomolecular interactions.

## 2. Materials and Methods

### 2.1. Chemicals and Materials

The powders of peptides P1 (HTYHEVTKH) and P2 (WPVLAYHF) were purchased commercially from Selleck Chemicals LLC (Houston, TX, USA), and their purity was higher than 95%. The stock solutions were prepared in phosphate buffer, and the concentration was 1·10^−3^ M. The phosphate-buffered saline (Sigma-Aldrich Chemie GmbH, St. Louis, MO, USA) was obtained by dissolving tablets in double distilled water, and the final pH was 7.4.

The plasma proteins: HSA (Sigma-Aldrich Chemie GmbH, St. Louis, MO, USA), AAG (Sigma-Aldrich Chemie GmbH, St. Louis, MO, USA), IgG (Sigma-Aldrich Chemie GmbH, St. Louis, MO, USA), and LYS (Sigma-Aldrich Chemie GmbH, St. Louis, MO, USA) stock solutions were prepared in phosphate buffer at a concentration of 1·10^−6^ M.

The stock solution of deoxyribonucleic acid sodium salt from calf thymus (ctDNA) (Sigma-Aldrich Chemie GmbH, St. Louis, MO, USA) was prepared in phosphate buffer with a concentration of 1 mg/mL. The actual concentration was determined spectrophotometrically per the procedure described by Hu and co-authors [22] and amounted to 6·10^−3^ M. The purity of the DNA preparation was assessed using the absorbance ratio at 260 and 280 nm. An A260/A280 value above 1.8 confirmed that the sample was essentially free of protein impurities. The concentrations of aqueous potassium iodide (KI) (Fluka, Honeywell Chemicals, Germany) and sodium chloride (NaCl) (Avantor Performance Materials Poland S.A., Gliwice, Poland) solutions were 1·10^−3^ M and 1 M, respectively. The following fluorescent dyes were used for the research: 4′,6-diamidine-2′-phenylindole dihydrochloride (DAPI) (Sigma-Aldrich Ltd., St. Louis, MO, USA), dansylated-L-glycine (dGly), dansylated-L-phenylalanine (dPhe) (Sigma-Aldrich Ltd., St. Louis, MO, USA), quinaldine red (QR) (Merck KGaA, Darmstadt, Germany). The fluorescence markers were dissolved in ethanol (Merck KGaA, Darmstadt, Germany) to obtain a 1·10^−3^ M concentration, and the solution was stored in the dark at 277 K.

### 2.2. The UV-Vis Measurements

#### 2.2.1. Plasma Protein Study

A Jasco V750 spectrophotometer (Jasco, Tokyo, Japan) was used to collect the absorption spectra. The UV spectra were collected in 10 mm path length quartz cuvettes at 298 K in the wavelength range 190–300 nm with a 0.2 nm interval. A baseline correction was applied, and the two accumulations were used to average each spectrum. Titration of the protein solutions (1.0·10^−6^ M) with the studied compounds, at various concentrations, was conducted to reach a final concentration of 5.5·10^−6^ M. The Benesi–Hildebrand procedure was used to calculate the K_a_ (apparent binding constant) [23]. The absorption peak was analyzed at a wavelength of 204 nm for HSA and 202 nm for AAG and LYS. This method of data analysis was employed as an approximate model assuming the formation of a dominant 1:1 peptide–biomacromolecule complex within the studied concentration range. It is worth noting that the constant K_a_, calculated using this approximate mathematical model, reflects the overall strength of the interaction under experimental conditions rather than absolute thermodynamic parameters. This approach made it possible to assume that the calculated binding constants determined for various proteins were sufficiently comparable. The plot of the absorbance changes and compound concentration is a linear relation that allows determining the constant K_a_ by fitting absorption UV data to the function (1) [24]:

(1) $$\frac{1}{A-A_{0}}=\;\frac{1}{A_{c}-A_{0}}+\frac{1}{K_{a}\left(A_{c}-A_{0}\right)c}$$

where A is the sample solution absorbance with different concentrations of the added component, A_0_ is the solution absorbance without the added component, A_c_ is the compound/biopolymer complex absorbance, and c is the added component concentration in each step, expressed in M. For the calculation of the standard free Gibbs energy (ΔG), Equation (2) was utilized:

∆*G* = −*RTlnK_a_* (2)

where R is the gas constant 8.314 Jmol^−1^K^−1^, T is the temperature 298 K, and K_a_ is the binding constant.

#### 2.2.2. The ctDNA Study

The UV-Vis absorption measurements of the DNA interaction were conducted on the same spectrophotometer as mentioned in Section 2.3.1. The apparatus settings were as follows: the wavelength range was 200–350 nm with a 0.2 nm data interval, and baseline correction was made. Each spectrum was obtained by averaging two consecutive scans recorded under identical conditions. The ctDNA solution was added to the samples containing the tested compounds. The final concentration of macromolecule was 5.2·10^−5^ M. The obtained data were subsequently analyzed in accordance with previously outlined, utilizing Equation (1) and then (2). The maximum of 202 nm band was selected for analysis.

### 2.3. The CD Measurements

#### 2.3.1. Plasma Protein Study

The circular dichroism (CD) spectra were recorded on the Jasco J-1500 magnetic circular dichroism spectrometer (JASCO International CO., Tokyo, Japan). The spectral range was 205–250 nm with a 0.2 nm step, and the scan speed was 100 nm/min with a 1 s response time. The measurements were conducted with a quartz 10 mm cuvette at 298 K (room temperature). The protein solutions: HSA, AAG, IgG, and LYS were prepared in phosphate buffer (pH = 7.5) at a 1·10^−6^ M concentration. The compound P1 or P2 was diluted in distilled water to achieve a 1·10^−3^ M concentration. Next, a small amount of P1 or P2 was added dropwise to the protein solution until a final concentration of 2·10^−6^ M. The spectra were recorded after each addition. The baseline correction was made, and five accumulations were used to accurately spectrum. After measurement, the recorded spectra were then analyzed using Jasco software to determine the percentage of secondary-structures in the protein. The analysis was performed using CD Multivariate Calibration Creation (version 2.02.00) and CD Multivariate SSE (version 2.03.00), based on multivariate chemometric analysis (PLS/PCR) using a reference protein CD spectral dataset. The reported percentages of secondary structure elements therefore represent model-derived estimates obtained from the experimental CD spectra rather than direct measurements of individual secondary-structure elements. The quality of the spectral fitting was assessed using the Root Mean Square Deviation (RMSD), with lower RMSD values indicating better agreement between the experimental and reconstructed spectra.

#### 2.3.2. The ctDNA Study

The measurements of the DNA interaction were carried out on the same apparatus with the following parameters: the wavelength range was 230–320 nm with a 0.2 nm data interval, the scan rate speed was 100 nm/min, and the response time of 1 s, three accumulations of each spectrum were made. The sample solution was prepared in a quartz cuvette with a 10 mm path length, and the measurements were conducted at 298 K (room temperature). The samples were prepared freshly by mixing phosphate-buffered solution with ctDNA stock solution, where the final concentration was 1·10^−4^ M. Subsequently, the compound P1 or P2 was added in stages to achieve a concentration of 1.64·10^−4^ M, after which the spectra for each mixture were recorded with baseline correction.

### 2.4. The Fluorescence Measurements

#### 2.4.1. Plasma Protein Study

The fluorescence spectroscopy measurements were performed by the Cary Eclipse 500 spectrophotometer (Agilent, Santa Clara, CA, USA). The fluorescence studies were carried out at 298 K in a quartz cuvette with a 10 mm path length.

In order to verify the binding site of compounds P1 and P2 in the selected proteins, the method of titration of protein complexes with a known marker by adding a solution of the tested compounds was used. The mixture of marker/protein was prepared with a final concentration in the sample of 1·10^−6^ M. Next, P1 or P2 was dropped into the solution, and after each addition, the spectrum was recorded. Different markers were used for each protein: DGly and DPhe for albumin, QR for α-1-acid glycoprotein. Before the displacement assay, the optimal excitation wavelength and emission range for the protein-fluorescence marker complexes were experimentally verified using the preScan function of the spectrofluorometer. The selected excitation wavelength ensured a high fluorescence signal intensity and an appropriate signal-to-noise ratio for monitoring ligand-induced displacement of the fluorescent probes. The excitation wavelength for dGly/HSA and dPhe/HSA complexes was 350 nm, and the emission range was 370–650 nm. The fluorescence parameters for the QR/AAG complex were as follows: the excitation wavelength was 500 nm, and the measured emission range was 520–750 nm. The percentage of marker exchange in the biomolecule complex was calculated by Equation (3):

(3) $$\%E=\frac{F_{0}-F}{F_{0}}\cdot 100\%$$

where %*E* is the percentage of replacement marker in the peptide–protein complex, F_0_ is the steady-state fluorescence intensity of the marker–protein complex, and F is the steady-state fluorescence intensity after compound P1 or P2 is added.

#### 2.4.2. The ctDNA Study

The experiments were carried out on the same apparatus as the tests with plasma proteins. The quartz cuvette with an optical 10 mm path length was used, and all measurements were lead in 298 K.

The studies with increasing iodide ion (addition of KI solution) concentration were made under two different conditions: the first one only with the studied compounds P1 or P2, and the second one with a mixture of the studied compounds P1 or P2 and ctDNA. The final concentration of compounds and/or ctDNA in the samples was 5·10^−5^ M. The samples were titrated with a solution of KI (1·10^−3^ M) in steps of 0.25 molar equivalents in the range of 0–2.0 molar ratios. The investigated compounds exhibited intrinsic fluorescence, characteristic of Tyr and Trp amino acid residues, and the optimal excitation wavelength was determined using the PreScan function of the spectrofluorometer. The fluorescence excitation wavelength was 280 nm, and emission spectra were measured in the wavelength range of 285–400 nm for P1 and 285–500 nm for P2. The determination of the Stern–Volmer (K_sv_) constants was calculated by Equation (4):

(4) $$\frac{F_{0}}{F}=1+k_{q}\tau \left[Q\right]=1+K_{\mathrm{SV}}\left[Q\right]$$

where F_0_ is the compound fluorescence intensity, F—compound fluorescence intensity with a quencher, k_q_ is the quenching rate constant, τ—the average fluorescence lifetime, K_sv_ is the Stern–Volmer constant, and [Q] is the quencher concentration.

Fluorescence intensity changes at different ionic strengths were studied by increasing the NaCl concentration from 0 to 0.112 M. The samples of P1 or P2 complexes with ctDNA (5·10^−5^ M) were titrated with NaCl (1 M) solution. The spectra were measured ten minutes after adding a drop of NaCl. The excitation parameters and emission range were selected based on the intrinsic fluorescence of the tested compounds. The excitation wavelength was 280 nm, and the emission spectra were measured in the range of 285–360 nm for P1 and 285–450 nm for P2.

The displacement of the fluorescent marker was performed by titration of the DAPI/ctDNA complex with P1 or P2 solutions. The 3 mL mixture of DAPI and ctDNA, whose final concentrations in the sample were 2.0·10^−6^ M and 4.0·10^−5^ M, respectively, was titrated with P1 or P2 compound solution. A small amount of P1 and P2 was added to the DAPI/ctDNA sample until the final concentration was reached (2.5·10^−5^ M). After each portion, the fluorescence spectra were recorded. The fluorescence excitation wavelength was 340 nm, and the emission spectra ranged from 360 to 600 nm. The fluorescence intensity was analyzed by Equation (3), and the value of decrease in DAPI–ctDNA fluorescence was determined.

### 2.5. The Molecular Docking

The crystal structures of HSA (2XW0 [25]), AAG (3KQ0 [26]), LYS (7XF6 [27]), and DNA (1VZK [28]) were downloaded from the RCSB Protein Data Bank. Proteins and DNA were prepared using ADFR v1.2 [29] software with the standard procedure. All the ligands and water molecules were removed, and then polar hydrogen atoms and Kollman charges were added to the protein and DNA structures. The grid box was set according to site I (center: x = 3.7, y = −11,0, z = 3.1, size: 16.5 × 13.5 × 20.3, spacing; 0.375) and site II (center: x = 9.5, y = 3.5, z = −15.5, size: 18.0 × 18.0 × 18.0, spacing; 0.375) for HSA, and for the entire structure for AAG (center: x = 27.9, y = −2.6, z = 2.1, size: 20.2 × 22.5 × 17.2, spacing; 0.375), LYS (center: x = −9.7, y = −13.7, z = 17.7, size: 26.2 × 29.2 × 25.5, spacing; 0.375), and DNA (center: x = 15.6, y = −20.9, z = 2.1, size: 26.2 × 23.3 × 41.3, spacing; 0.375). Geometry optimization of peptides was done using DFT with B3LYP/6-31+G (d.p) basis set. Optimizations were performed assuming the peptides at physiological pH. The charge of both compounds were −1. Computation was performed using the Gaussian 2016 C.01 software package. This was the starting conformation for docking. To prepare the peptides, partial charges were calculated, nonpolar hydrogens were merged, and rotatable bonds were assigned. AutoDock CrankPep (ADCP [29], version 1.1) software was used for docking. Parameter N (runs) was set to 100 with 2,000,000 evaluations (n). The clustering criteria and ranking procedure are described in detail in [29]. The docking protocol was validated by redocking the co-crystallized ligands. The Root Mean Square Deviation (RMSD) values were calculated as follows: 0.577 Å for DNA, 1.210 Å for site I of HSA, 0.464 Å for site II of HSA, 1.105 Å for AAG. Due to the absence of the native ligand in the crystallographic structure, validation for LYS was not performed. The visualizations were done using Biovia Discovery Studio Visualizer v.24.1 (https://discover.3ds.com/discovery-studio-visualizer-download, accessed on 12 January 2026). The pharmacophore model was generated using the Pharmit server (accessed on 12 January 2026) [30].

## 3. Results and Discussion

### 3.1. Interaction with Blood Serum Proteins

One of the stages in discovering the distribution, metabolism, and efficacy of bioactive compounds is binding studies with blood plasma proteins. The main protein is albumin because its concentration in blood is approximately 60% [24,31]. However, other proteins, such as immunoglobulins, glycoproteins, or lipoproteins, also play a crucial role in the distribution of small molecules in the body. Including different transport biomolecules in such studies allows a broader view of how active compounds are distributed in the body. We therefore decided to test the analyzed peptides for their interaction with four plasma proteins: HSA, AAG, LYS, and (IgG). This is a broader and more comprehensive approach that has been gaining ground recently. By considering interactions with four plasma proteins differing in structure and physiological function, rather than HSA alone, this perspective provides a more representative view of the plasma environment, thereby establishing a more reliable basis for predicting the potential absorption, transport, distribution, and pharmacokinetic behavior of the investigated compounds in vivo. Moreover, the present study extends the previously reported biological evaluation of similar compounds by providing physicochemical information on their interactions with several physiologically relevant plasma proteins.

**Figure 1:**
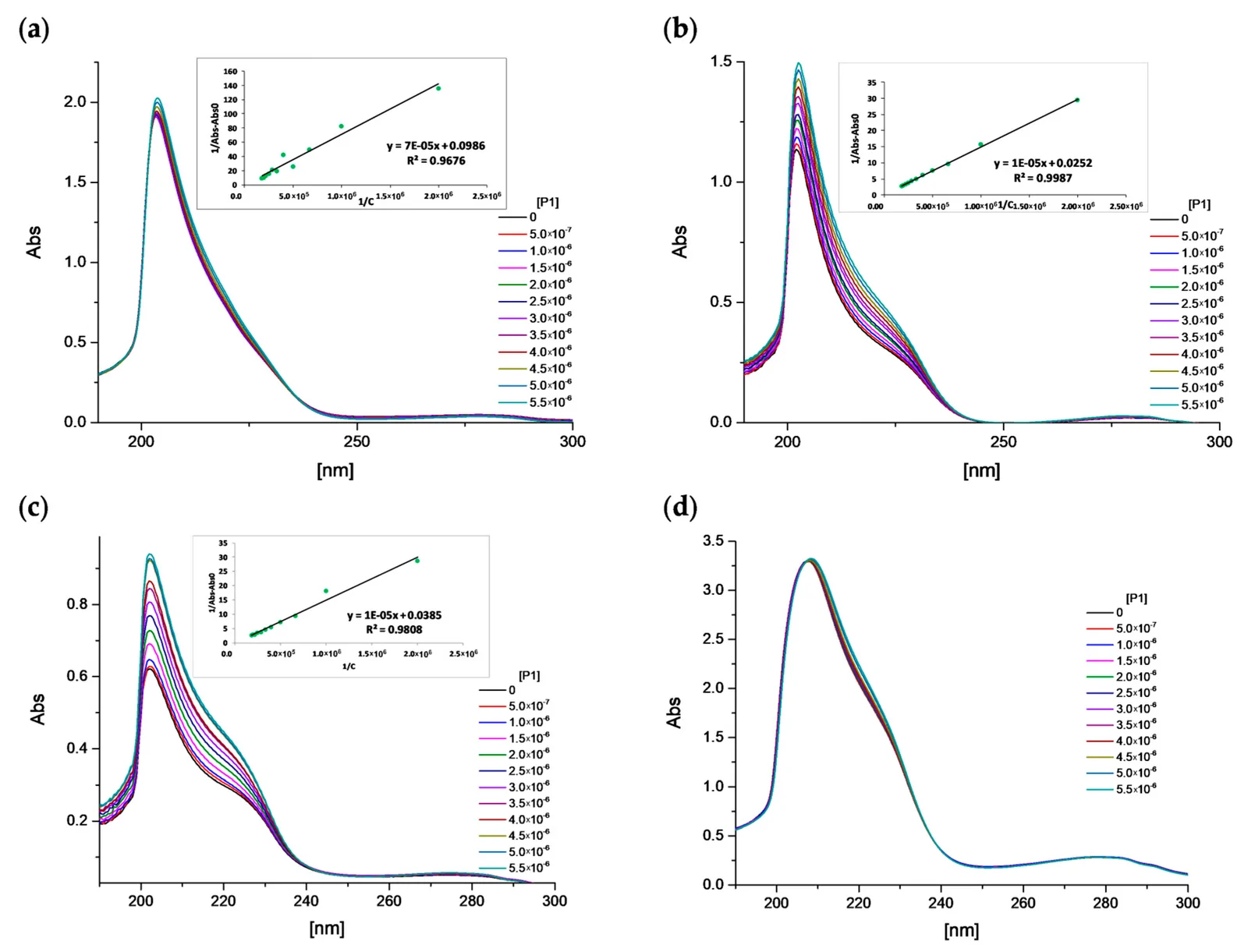
The UV spectra with Benesi–Hildebrand plots of the addition of P1 to protein solutions: (**a**) HSA, (**b**) AAG, (**c**) LYS, (**d**) IgG.

**Figure 2:**
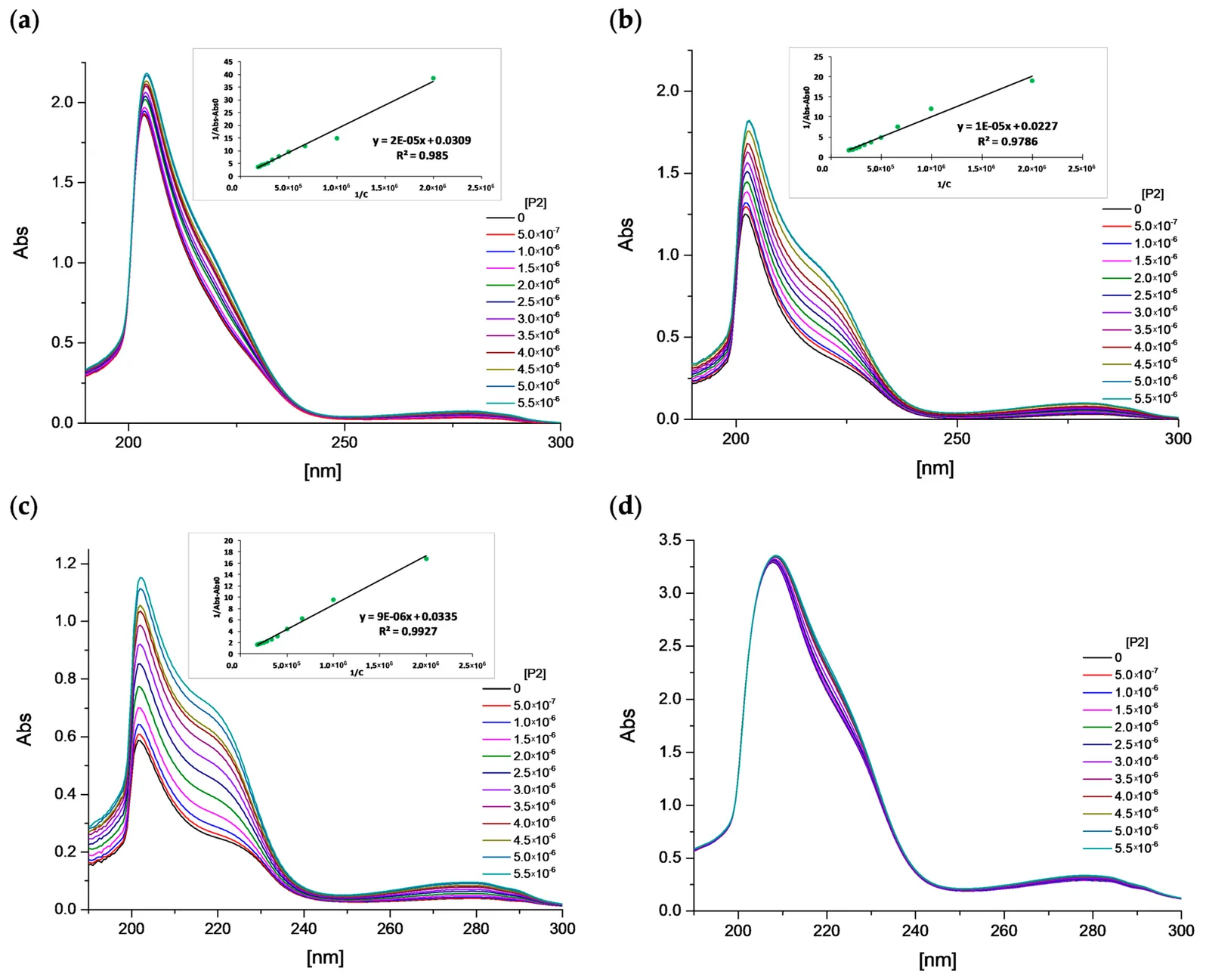
The UV-Vis spectra with Benesi–Hildebrand plots of the addition of P2 to protein solutions: (**a**) HSA, (**b**) AAG, (**c**) LYS, (**d**) IgG.

The UV absorption analyses confirm the formation of complexes between the proteins and the studied compounds. In all cases, the addition of compounds to protein solutions led to a hyperchromic effect (Figure 1). However, the magnitude of these changes and their dependence on protein concentration were insufficient to allow reliable determination of the association constant using the Benesi–Hildebrand approach under the experimental conditions employed. Therefore, the absence of a reliable association constant for IgG should not be interpreted as evidence for the absence of binding, but rather as a limitation of the applied spectrophotometric approach and concentration range. For the remaining proteins in the tested concentration range, a linear increase in the degree of absorption was observed, allowing the straight-line equation to be fitted according to the Benesi–Hildebrand procedure (Figure 1 and Figure 2) [23,24]. The values of the binding constants (K_a_) are summarized in Table 1. The highest values were obtained for the interaction of P1 and P2 with lysozyme, slightly lower with acid glycoprotein, and the lowest for albumin. The binding constants were on the order of 10^3^ M^−1^. The negative ΔG values indicate that association is thermodynamically favorable under the experimental conditions. Analogous values were also obtained for the interactions of glutathione with bovine albumin (20·10^3^ M^−1^) and L-ornithine with human albumin (1.9·10^3^ M^−1^) [32,33].

Furthermore, the protein–protein interaction between the proteolytic enzyme trypsin and human albumin is determined by a binding constant equal to 31·10^3^ M^−1^ [34]. The standard Gibbs free energy (ΔG) reaches negative values (Table 1). The negative ΔG values indicate thermodynamically favorable peptide–protein association under the experimental conditions. These interactions may potentially influence the free peptide fraction, systemic transport, and distribution, and could therefore be relevant to the biological availability of P1 and P2. However, the present study did not assess pharmacokinetic parameters, proteolytic stability, cellular uptake, or in vivo bioavailability; therefore, these possible consequences should be regarded as hypotheses requiring further experimental validation.

**Figure 3:**
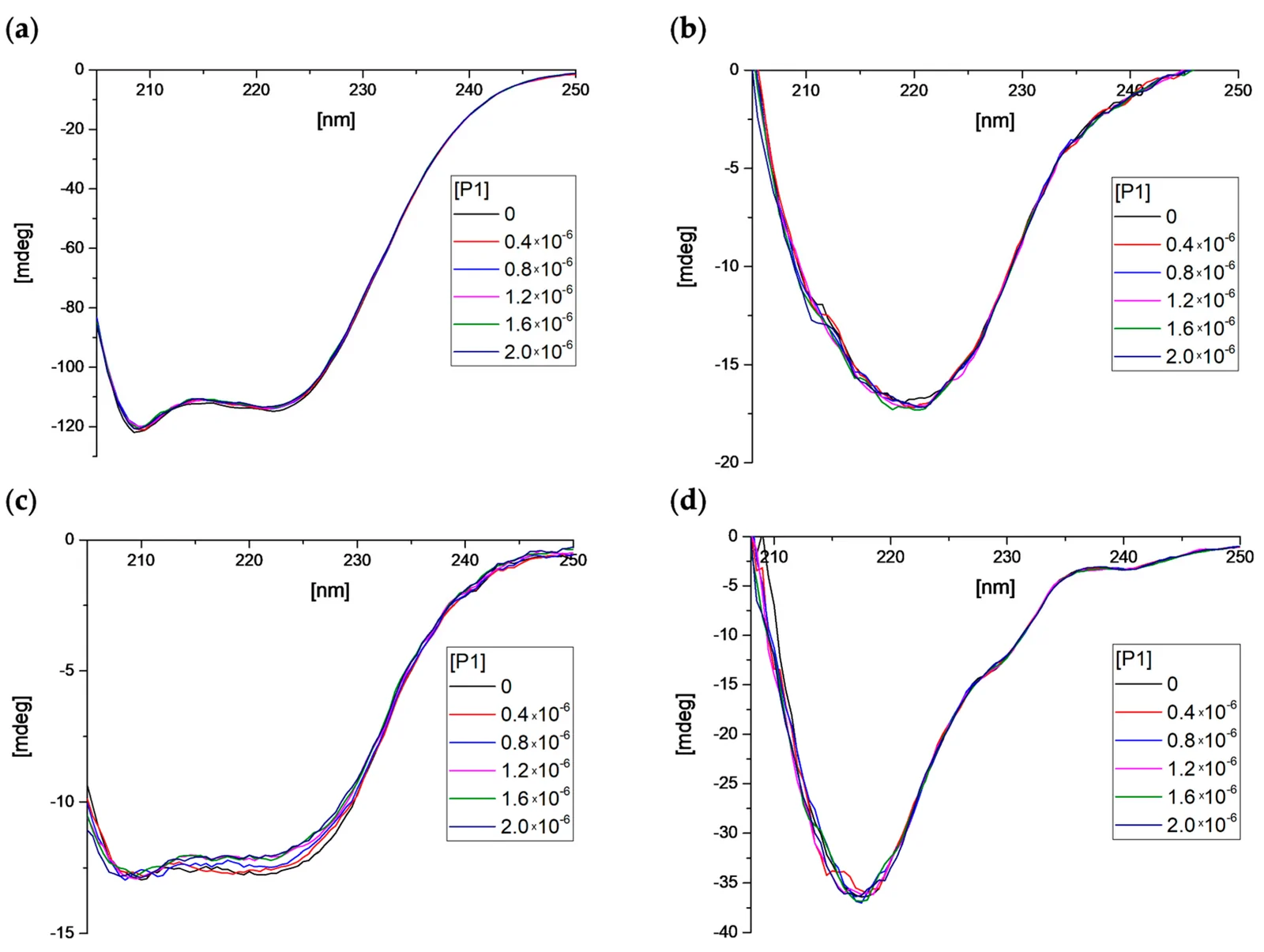
The CD spectra for P1 with: (**a**) HSA, (**b**)AAG, (**c**) LYS, (**d**) IgG.

**Figure 4:**
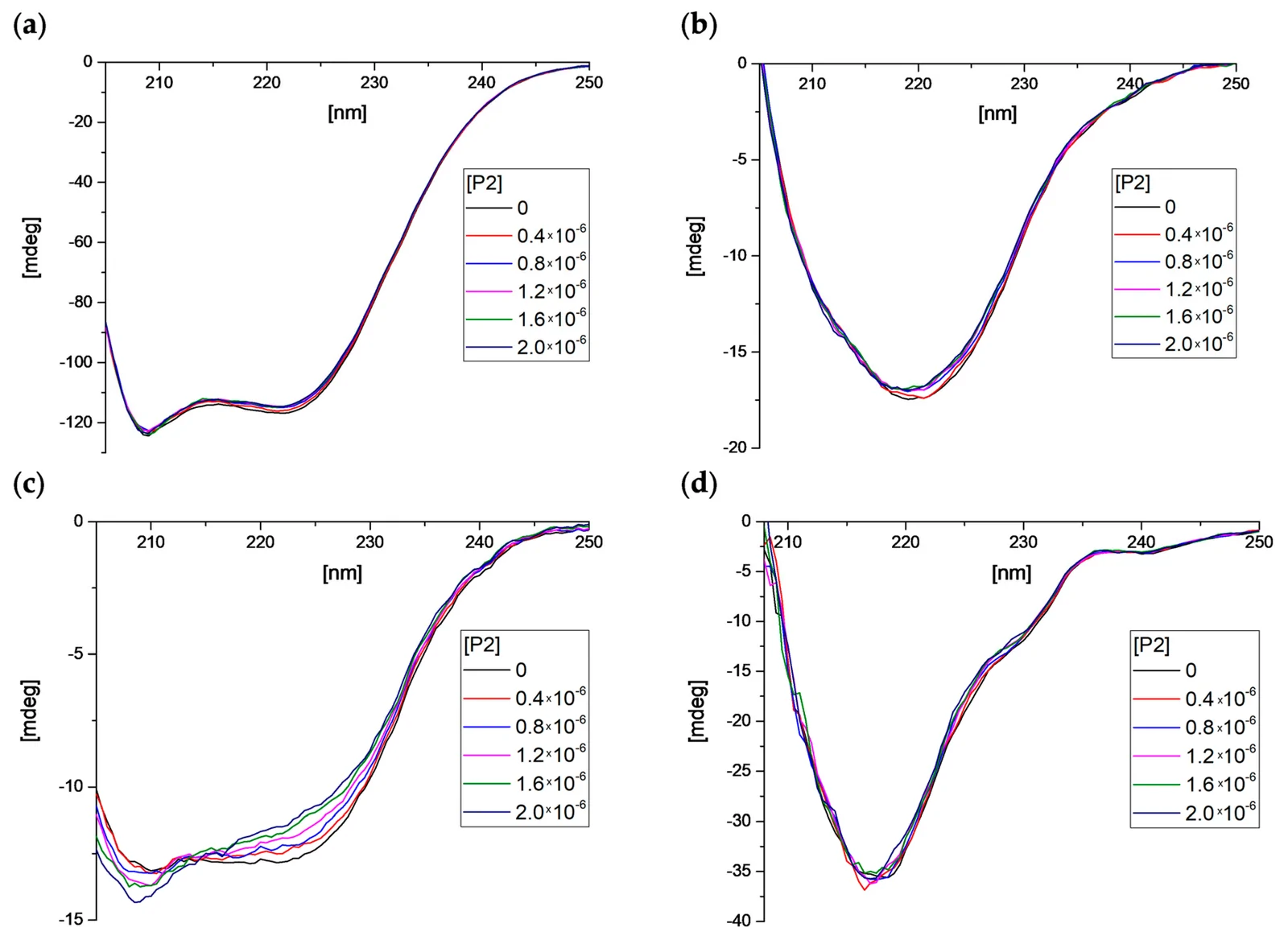
The CD spectra for P2 with: (**a**) HSA, (**b**)AAG, (**c**) LYS, (**d**) IgG.

In order to analyze the effect of the binding of the tested peptides on the secondary structure of plasma proteins, circular dichroism spectroscopy was employed. The spectra obtained for the tested proteins and complexes with peptides P1 and P2 are shown in Figure 3 and Figure 4. Each reported spectrum represents the average of five consecutive scans recorded under identical conditions. This commonly used acquisition procedure was applied to improve the signal-to-noise ratio and verify the short-term stability of the spectral signal.

All recorded spectra are characteristic of the secondary structure of the examined proteins [35,36,37,38,39,40]. The obtained results showed that only minor changes in the secondary-structure profiles of the studied macromolecules were observed upon complex formation with P1 and P2 under the experimental conditions. With the increase in the concentration of the analyzed peptides, the position of the bands in the spectra remains unchanged. In the case of lysozyme, minor changes in the intensity of the observed signals can be observed. In order to more precisely analyze the obtained results, the percentage of individual secondary forms of proteins was calculated (Table 2, Table 3, Table 4 and Table 5).

In the absence of peptides, HSA displays a characteristic structure that is predominantly composed of α-helices, with minor contributions from β-sheets, β-turns, and unordered structures (Table 2). Upon binding of peptide P1, a modest decline in α-helical content was discerned. This was accompanied by a modest increase in β-sheet content. The β-turn and random coil fractions remained largely unchanged. A similar situation can be observed for the P2 compound. The results obtained from this study suggest that both peptides interact with HSA, inducing subtle conformational changes, primarily characterized by a small loss of α-helical structure and a minor gain in β-sheet content. The overall structural integrity of HSA is maintained, suggesting that peptide binding does not cause unfolding or denaturation of the protein.

The secondary structure of orosomucoid was also examined in the absence and presence of two peptides, P1 and P2, at concentrations of 0.8∙10^−6^ and 2.0∙10^−6^ M (Table 3). The results suggest that both peptides interact weakly with AAG, inducing subtle concentration-dependent shifts in the secondary structure. Peptide P1 appears to slightly destabilize β-sheet regions, while peptide P2 causes a slight increase in both α-helical and β-sheet content, possibly due to stabilization or slight ordering of the peptide–protein complex. It is evident that the structural integrity of α1-acid glycoprotein is largely preserved, with only minor conformational disruptions observed upon peptide binding. This finding suggests a moderate interaction with localized structural effects, rather than global unfolding.

Peptides P1 and P2 induce slight conformational rearrangements in lysozyme, primarily reflected by a gradual reduction in α-helical content and a minor increase in random coil and β-turn structures (Table 4). These subtle changes indicate partial structural relaxation or surface-level interactions, without major disruption of the overall protein fold. In consideration of the recognized stability of lysozyme, which is a globular enzyme characterized by its well-packed tertiary structure, these minor secondary-structure changes are consistent with non-denaturing interactions between the peptides and the protein.

The secondary-structure analysis of IgG in the absence and presence of peptides P1 and P2 reveals minimal changes upon complex formation. In all samples, the α-helix content remains low (~6.4–6.7%), with β-sheet structures dominating due to the immunoglobulin fold. These findings suggest that only minor conformational changes in the secondary structure of IgG were observed upon binding of P1 and P2 at the tested concentrations.

Overall, the data show that both peptides exert only minor effects on the secondary structure of the proteins. These results suggest that the analyzed compounds form complexes with the proteins without substantially disrupting their spatial structure.

**Figure 5:**
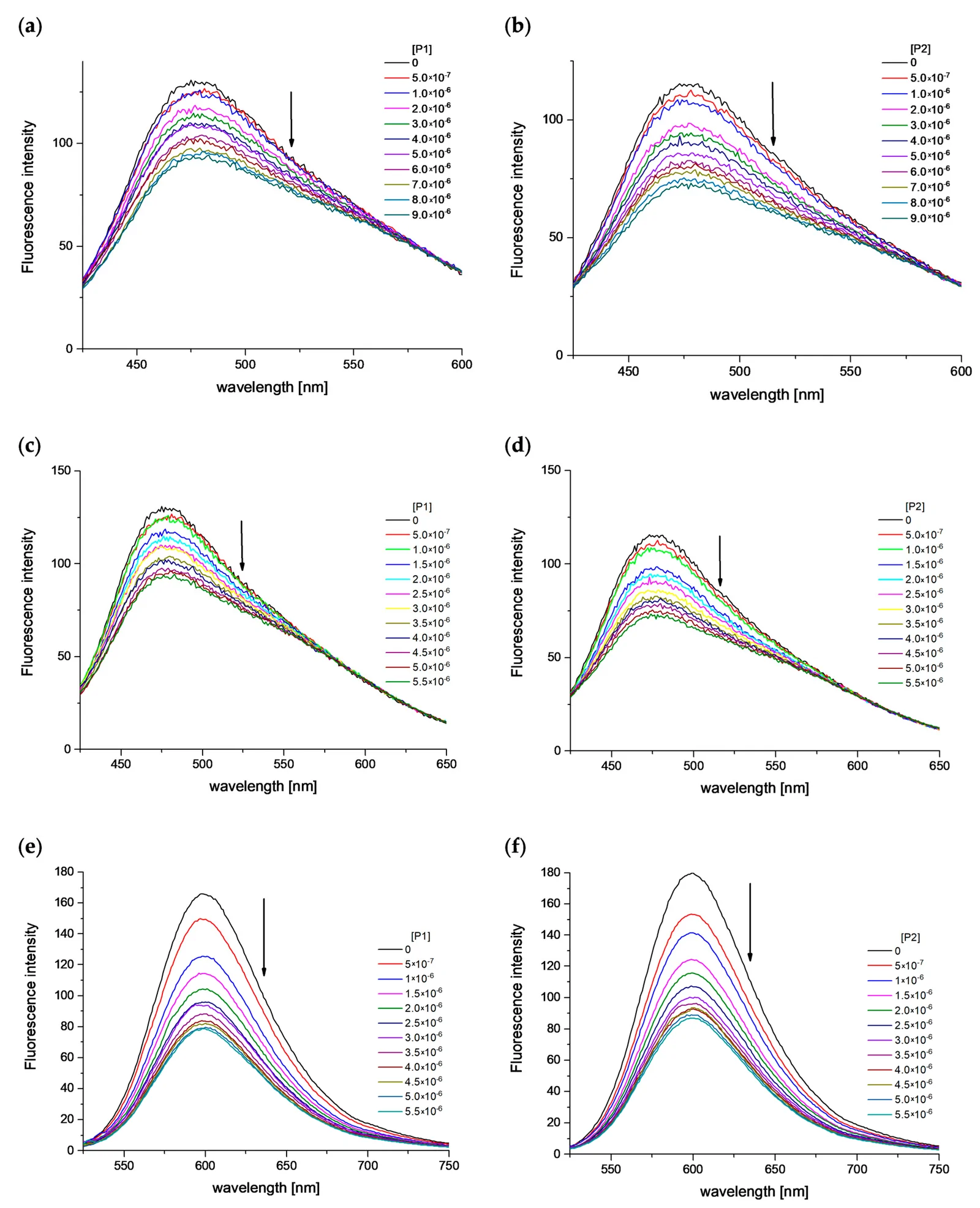
The fluorescence spectra of: (**a**) dGly/HSA complex titrated by compound P1, (**b**) dGly/HSA complex titrated by compound P2, (**c**) dPhe/HSA complex titrated by compound P1, (**d**) dPhe/HSA complex titrated by compound P2, (**e**) QR/AAG complex titrated by compound P1, (**f**) QR/AAG complex titrated by compound P2. The arrow indicates the direction of the change in fluorescence intensity observed with increasing concentration of the peptide under study.

In the subsequent stage of the investigation, the potential for the fluorescent marker to be expelled from the binding site within the protein was examined. A significant number of binding sites in albumin have been characterized for various substances, including drugs, fatty acids, and metal ions. The primary site of interaction with drugs may be the Sudlow site I (subdomain IIA in HSA) or the Sudlow site II (subdomain IIIA in HSA) [5,41,42]. Site I is a relatively large hydrophobic cavity, in which the aromatic rings or heterocyclic anions (e.g., warfarin) of the ligands are anchored by hydrophobic protein residues. In contrast, Site II is characterized by a smaller size than Site I and hydrophobic properties. However, its geometry and residue arrangement differ from those of Site I, thereby influencing the preferences of other ligands. Sudlow’s site II is characterized by the presence of a nonpolar region [41]. This pocket is known to be favored by aromatic carboxylates. The model ligand for this cavity is the nonsteroidal anti-inflammatory drug ibuprofen [41]. In this study, dGly, which prefers Sudlow site I, and dPhe were used to label Sudlow site II as markers of specific binding sites in HSA [43]. The excitation wavelength was set at 350 nm, corresponding to the absorption maximum of the dansyl fluorophore. Dansyl derivatives exhibit strong absorption in the 330–360 nm range and a high fluorescence quantum yield, particularly upon transfer to the hydrophobic environment of the albumin binding pockets [42,44]. Consequently, excitation at 350 nm has been found to provide the most efficient emission spectra of both dGly and dPhe, while concomitantly minimizing interference from the intrinsic fluorescence of human serum albumin, which arises principally from the Trp-214 residue and is optimally excited at approximately 280 nm. The incorporation of compounds P1 and P2 into the solution comprising the dGly/HSA and dPhe/HSA complexes led to a reduction in band intensity (see Figure 5). The percentages of ligand exchange, calculated using Equation (3), are presented in Table 6. A reduction in fluorescence of approximately 30% is observed in all four systems studied. The obtained result suggests either competition between the two markers or an alteration of the tracer’s environment. Assuming the first option, it can be stated that it is probable that both peptides bind to two binding sites in the albumin molecule. However, based on the experiment performed, it is difficult to determine the affinity for the binding pockets. With regard to the second protein under analysis, AAG, it should be noted that, despite the presence of multiple binding sites, only a single one is of clinical significance in the context of drug transport. QR is a characteristic dye to confirm this binding site [45,46]. QR is a highly conjugated cationic fluorophore characterized by strong absorption in the green spectral region and a broad emission band extending into the red region of the spectrum. Its high molar absorptivity and efficient fluorescence make it a suitable probe for monitoring micro-environmental changes in acidic glycoprotein matrices [47]. The obtained fluorescence spectra for both systems tested in this context, with increasing concentration of the tested peptides, are presented in Figure 5e,f, whereas the percentage of fluorescence exchanged dye is summarized in Table 6. The obtained results indicate that both of the peptides under consideration have been confirmed to occupy the position in the protein that is occupied by the marker. This outcome has been found to result in an exchange percentage of 53% for P1 and 52.2% for P2.

**Figure 6:**
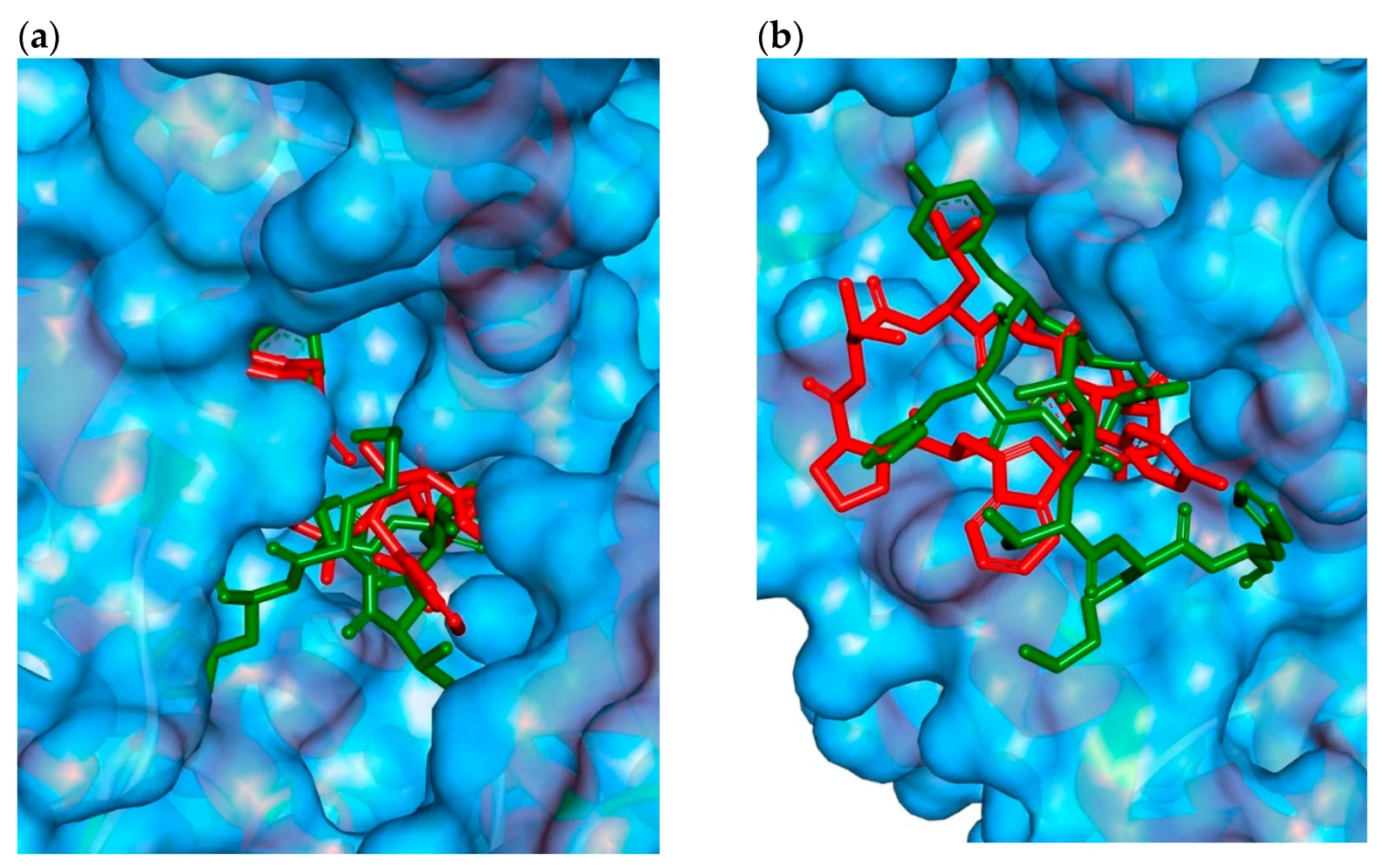
Binding poses of peptides P1 (green) and P2 (red) in: (**a**) HSA siteI and (**b**) site II.

**Figure 7:**
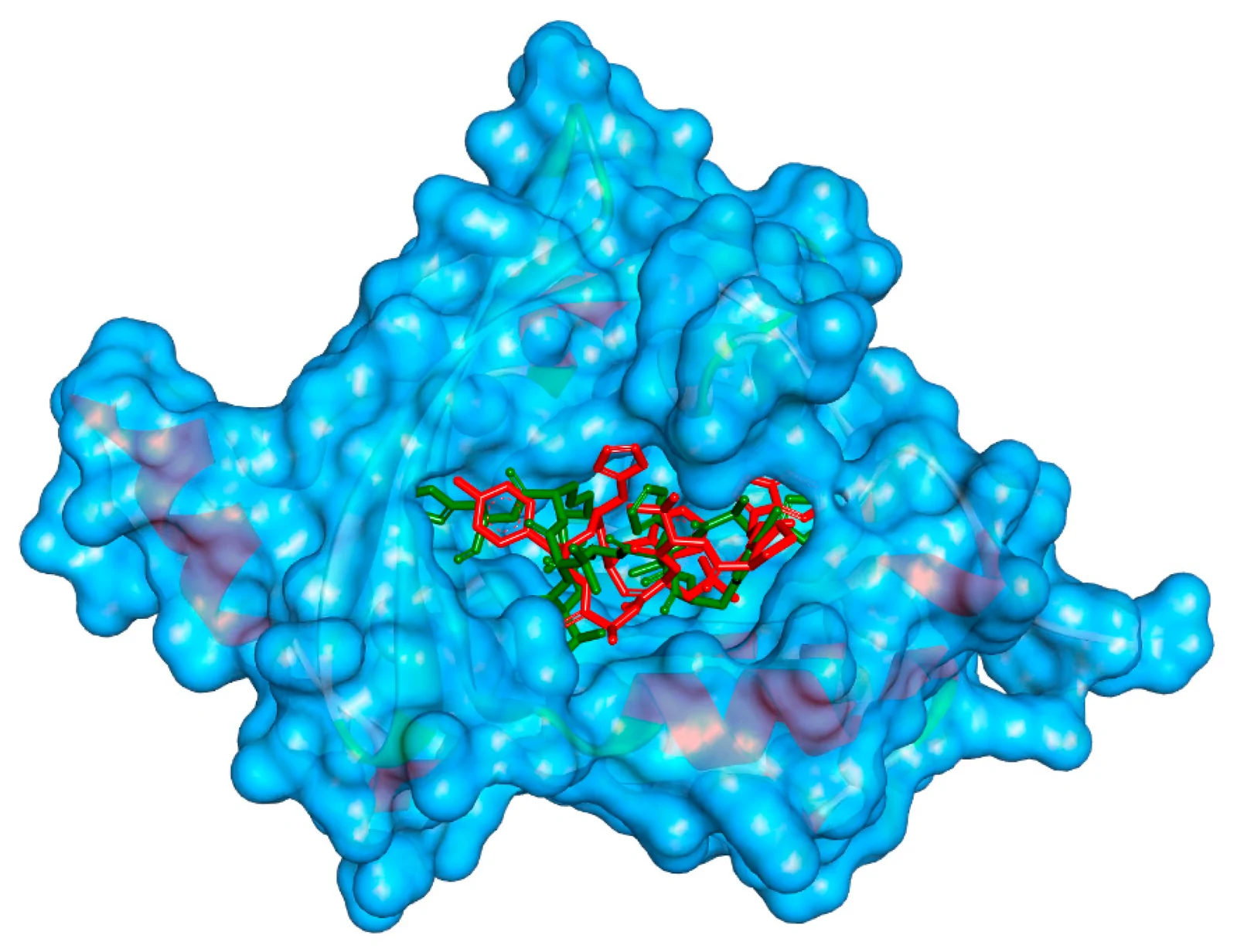
Binding poses of peptides P1 (green) and P2 (red) in AAG.

**Figure 8:**
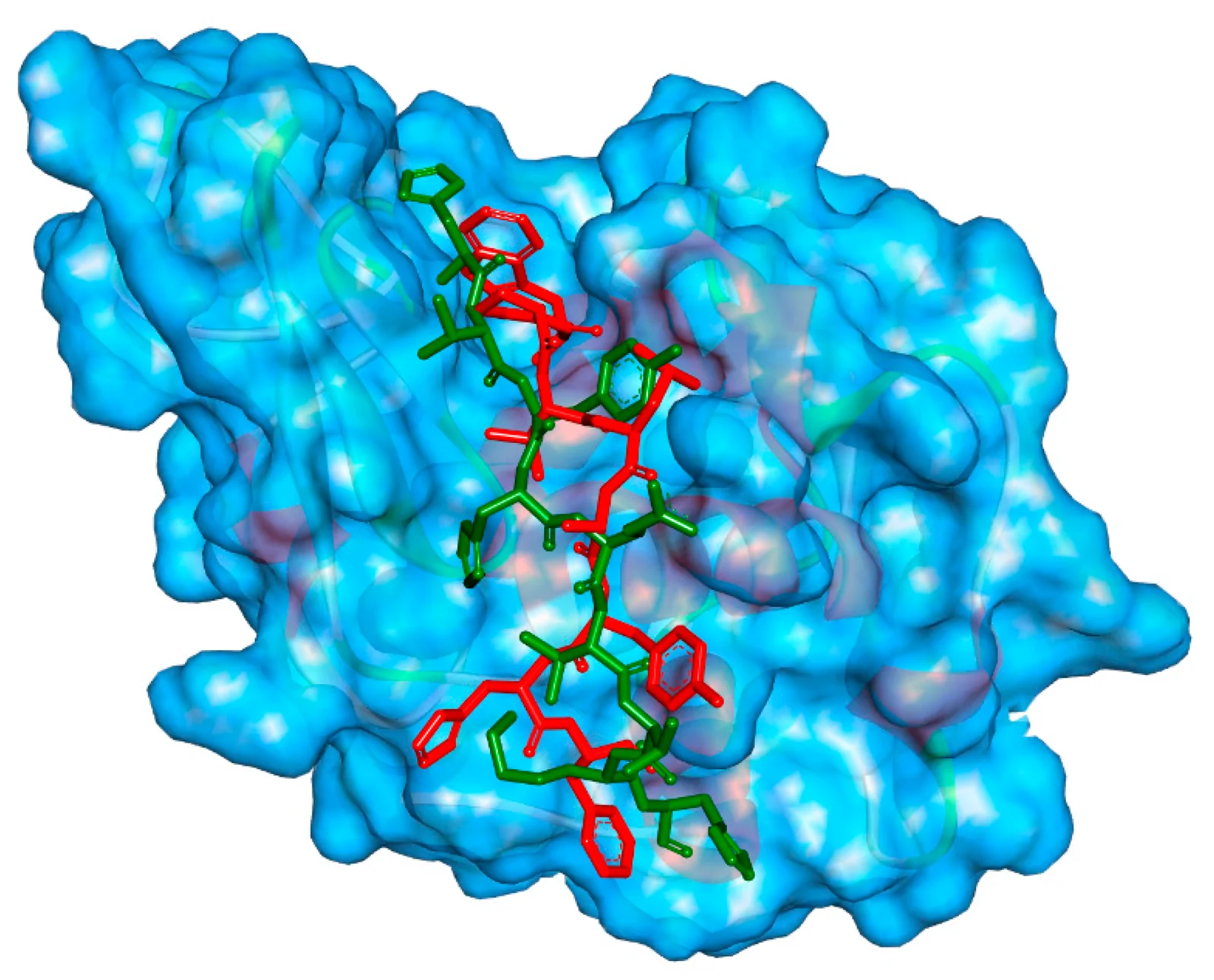
Poses of peptides P1 (green) and P2 (red) in lysozyme (LYS).

Concerning spectroscopic studies, docking to HSA was performed at Sudlow’s drug binding sites: site I (IIA) and site II (IIIA). The docking scores were −16.9 and −14.9 kcal/mol for P1 at sites I and II, respectively, and −16.1 and −16.7 kcal/mol for P2. These values represent scoring-function estimates used for the relative assessment of the predicted binding poses and should not be interpreted as experimental binding affinities or measures of complex stability. Both peptides adopt a binding pose that allows them to partially interact with HSA at site I or II (inside), while the remaining part of the molecule interacts with the surface of HSA (Figure 6). In the case of site I, which is larger, the peptide is able to penetrate deeper into the pocket. At site I, P1 interacts with HSA via hydrogen bonds involving the amino acid residues H1, T2, Y3, H4, and V6 (Figure S1). Peptide P2 forms hydrogen bonds through V3 and F8. The predicted poses also involve several hydrophobic interactions, including π-anion, π-sigma, π-cation, π-donor, π-alkyl, alkyl, and π-π T-shaped interactions (Figure S2). At site II, peptide P1 interacts via hydrogen bonds with H1, T2, K8, and H9 (Figure S3). Peptide P2 forms hydrogen bonds via W1, A5, Y6, and H7 (Figure S4). Hydrophobic interactions are also present in the predicted poses. For the analysis of interactions with AAG and LYS, molecular docking was performed using the entire protein structure The docking scores were −17.8 and −18.4 kcal/mol for P1 and P2 with AAG, respectively, and −17.1 and −15.7 kcal/mol for P1 and P2 with LYS, respectively. Both compounds were predicted to interact with AAG in the entry pocket region (Figure 7). In the case of P1, hydrogen bonds with the protein are formed with the amino acids T2, E5, V6, and K8, whereas P2 forms hydrogen bonds with Y6 and H7. The predicted poses also involve various hydrophobic interactions (Figures S5 and S6). For the lysozyme systems, both tested peptides bind within the groove between the two domains, α-helix and β-sheet domains (Figure 8), where the predicted poses involve numerous hydrogen-bonding interactions. For P1, hydrogen bonds are formed with K51, E53, S54, G55, N62, N64, D71, R133, and N132 amino acid residues of lysozyme. For the system with P2, hydrogen bonds involve K51, W52, D71, A129, and R133. Hydrophobic interactions are also present in the predicted complexes (Figures S7 and S8).

**Figure 9:**
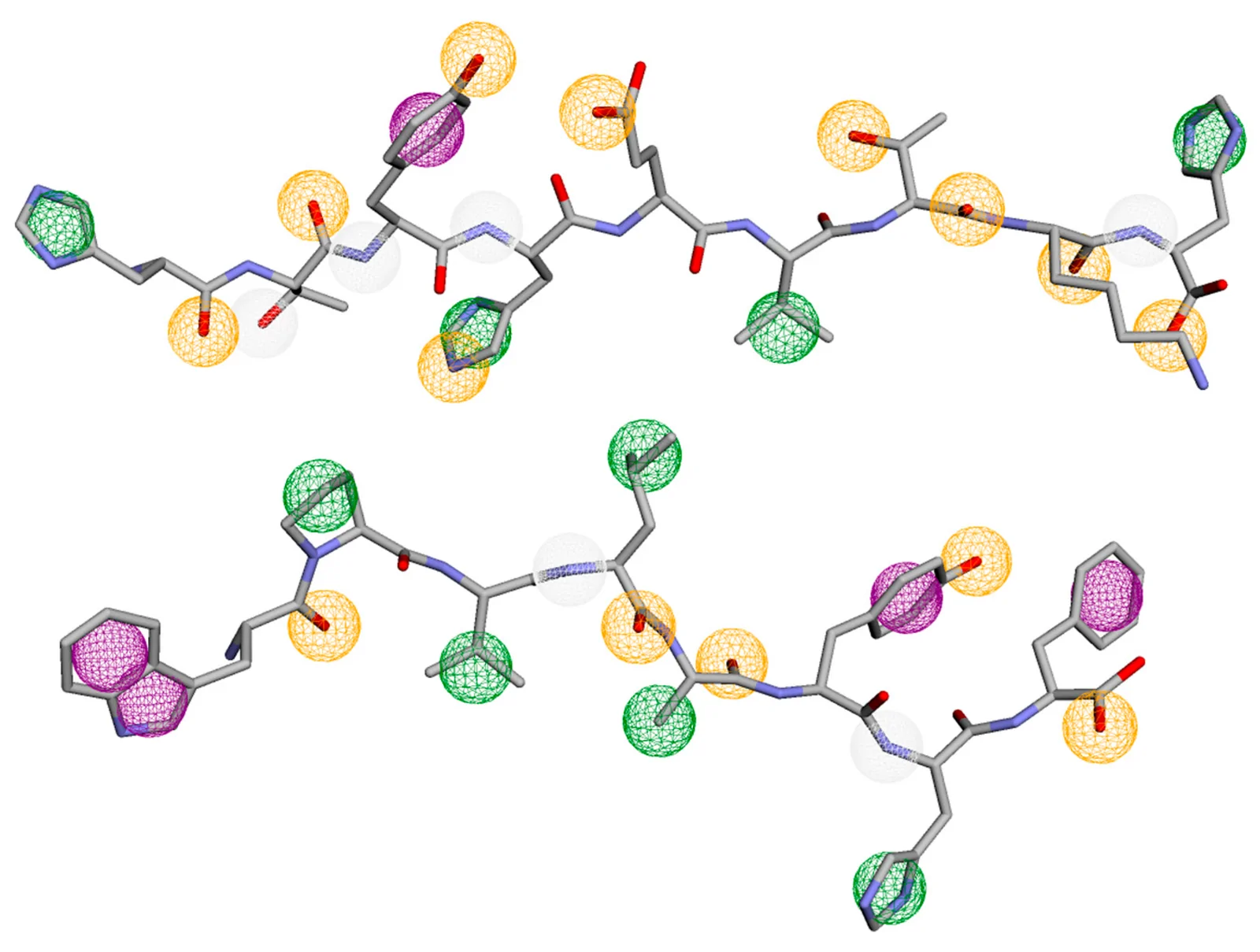
Pharmacophore models of peptides P1 (**top**) and P2 (**bottom**) were generated based on molecular docking analysis. The model includes hydrogen bond donors (light grey) and acceptors (orange), hydrophobic regions (green), and aromatic (purple) moieties corresponding to the key interactions observed in protein–peptide complexes.

Based on the key interactions identified from molecular docking analysis, pharmacophore models for both compounds were proposed (see Figure 9). The model reflects the interaction pattern observed across protein systems, including HSA, AAG, and LYS. For peptide P1, the model highlights the crucial interactions observed in the protein–peptide complexes, particularly those involving residues H1, T2, Y3, and V6, engaged in interactions with almost all proteins. The pharmacophore comprises hydrogen bond donor and acceptor features corresponding to the peptide backbone and side-chain functionalities, which are responsible for stable complex formation through interactions with polar and charged residues. The model also includes features corresponding to interactions involving π-systems. These interactions are mainly associated with residues such as H1 and Y3. Overall, the P1 pharmacophore is characterized by a balanced contribution of hydrogen-bonding and π-type interactions. In contrast, the pharmacophore model for peptide P2 reveals a slightly different interaction pattern. The stabilization of complexes in this case is more strongly influenced by hydrophobic and π-type interactions. The final model comprises a limited number of key features, including hydrogen bond donor and acceptor sites, hydrophobic regions, and aromatic moieties, which together define the minimal structural requirements for effective binding.

In summary, the combination of spectroscopic studies and molecular modeling demonstrated that both analyzed peptides interact with the plasma proteins under investigation. The observed protein binding may influence the distribution profile of the peptides. However, dedicated pharmacokinetic studies are required to determine whether these interactions translate into altered circulation time, bioavailability, or tissue distribution in vivo. The obtained association constants (~10^3^ M^−1^) indicate relatively weak, non-covalent peptide–protein interaction under the experimental conditions. Excessively weak affinity for plasma proteins could lead to rapid elimination and high concentrations of the free fraction in the bloodstream, potentially increasing side effects, whereas excessively strong binding would reduce the biologically active fraction. Conversely, a moderate degree of binding facilitates a favorable balance between systemic transport, protection against degradation, and effective release at the target site. Thus, the process of binding to proteins may positively impact the ADME profile of the peptides, supporting their distribution while maintaining bioavailability [48,49,50]. However, to confirm this hypothesis for the peptides under study, a series of additional in vitro and in vivo experiments would need to be conducted.

### 3.2. Interaction with ctDNA

**Figure 10:**
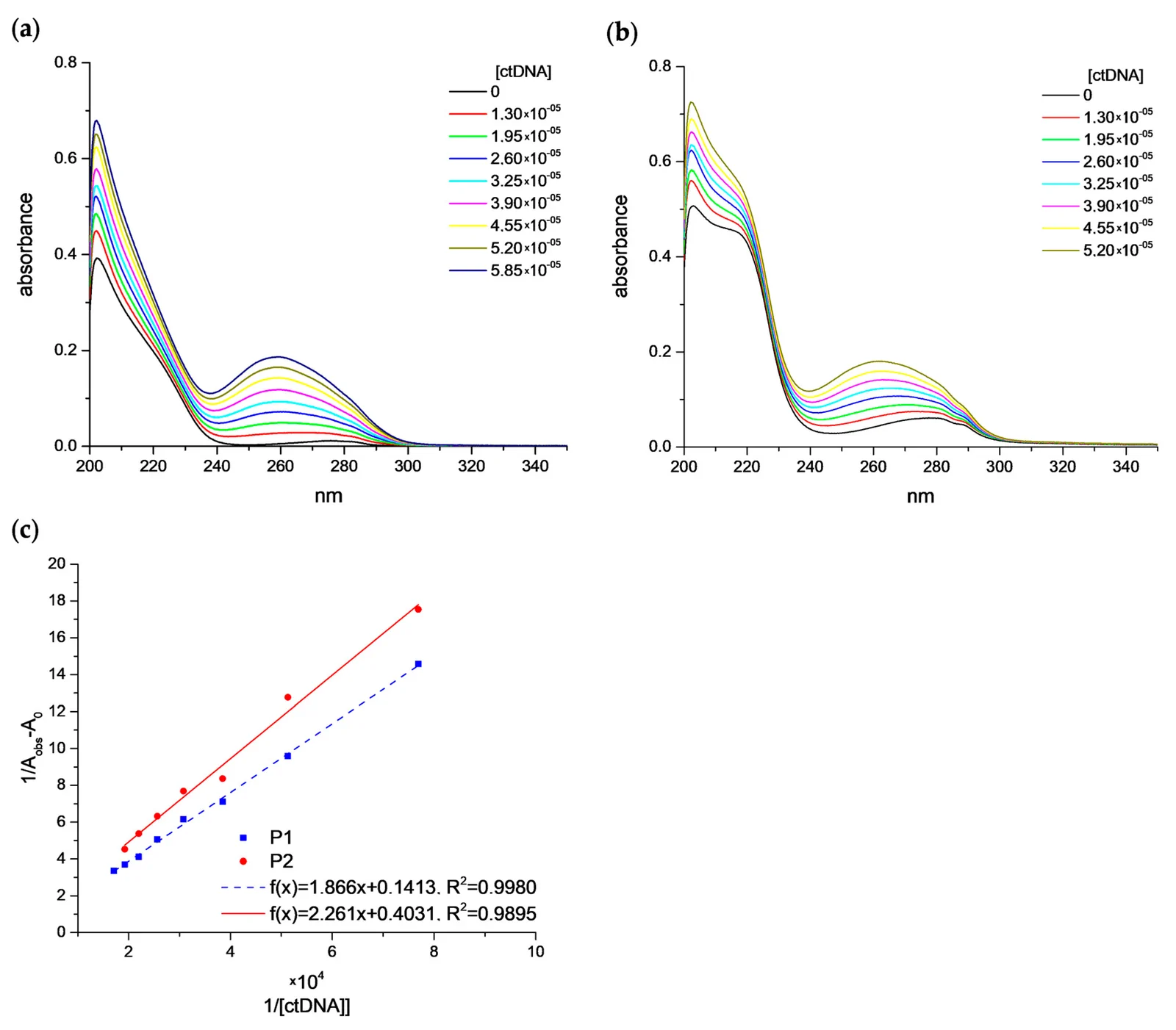
The UV-Vis spectra of compounds (**a**) P1 (square mark and blue slight line) and (**b**) P2 (round mark and red dashed line) by the addition of ctDNA solution. (**c**) The analysis of data by the Benesi–Hilderbrand method.

The interaction between bioactive compounds and DNA molecules has been the focus of extensive research in recent years. The combination of spectroscopic methods allows for an in-depth description of the binding mechanisms, the strength of this interaction, and the influence on the spatial structure of biomolecules. The evaluation of the thermodynamic parameters, including the binding constants (K_a_) and the standard free Gibbs energy (ΔG), was conducted through the analysis of UV absorption spectra. The calf thymus DNA (ctDNA) occurs in the B-form and possesses a standard double-stranded structure [51,52,53]. The absorption spectra of ctDNA exhibited two maxima at 200 and 258 nm (Figure 6a,b). The first band was then subjected to a detailed analysis in a binding study. The incorporation of a small quantity of P1 or P2 into the ctDNA solution gives rise to hyperchromicity of the absorption band equal to 71.00% for P1 and 43.94% for P2 (Table 7) and is accompanied by a slight 3 nm red shift. The complex formation was observed and analyzed by the Benesi–Hildebrand method [23]. The plot of the absorbance changes and compound concentration is a linear relation (Figure 10c). The binding constant (K_a_) is found to be 0.75·10^3^ M^−1^ for P1, while for compound P2, it is determined to be 1.78·10^3^ M^−1^ (Table 7).

The calculated ΔG by Equation (2) is collected in Table 7, and negative values indicate that the process of forming a complex between the tested compounds and the ctDNA molecule is a spontaneous reaction under standard conditions.

**Figure 11:**
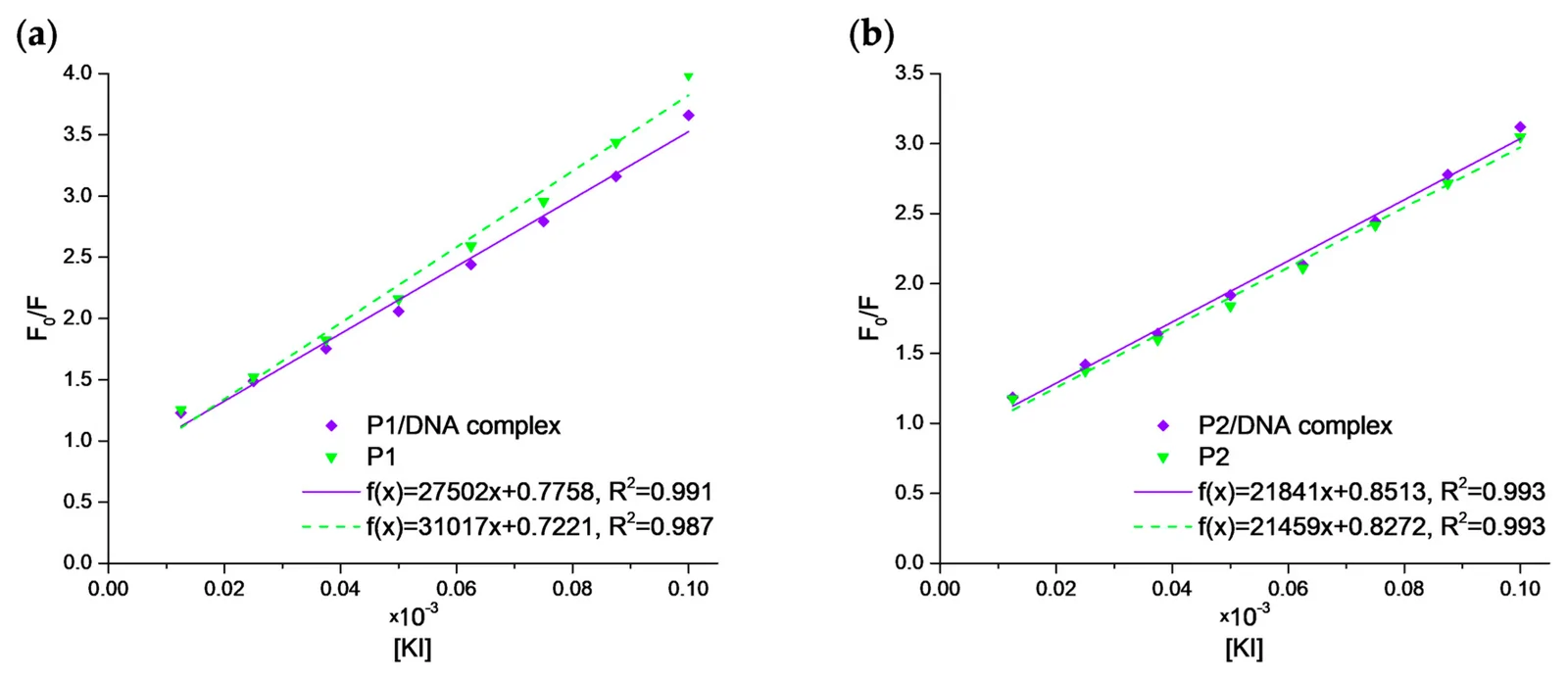
The analysis of fluorescence spectra of titration: (**a**) P1 with ctDNA (purple mark and straight line) and without ctDNA (green mark and dashed line) by KI solution, and (**b**) P2 with ctDNA (purple mark and straight line) and without ctDNA (green mark and dashed line) by KI solution.

The subsequent stage in the series of DNA binding studies was the evaluation of Stern–Volmer constants (K_SV_) with a known quencher, potassium iodide. The observation of changes in fluorescence intensity with increasing I^−^ ion concentration enables the assessment of whether a given compound binds to DNA in the groove or intercalation manner. The groove binding agent is exposed to the action of the iodide ion, thus facilitating straightforward quenching. Whereas the intercalative compound in complex with ctDNA is protected by the double-strand structure from the quenching effect of the iodide ion [54,55]. However, these observations should be regarded as indirect evidence and interpreted together with the results obtained from other experimental and computational techniques. In this study the Stern–Volmer constants (K_SV_) were compared for two samples: one comprised compounds only (excluding ctDNA), and the other comprised complexes with ctDNA. In both systems, a decrease in fluorescence intensity was observed as the KI concentration increased. The Stern–Volmer plot is a linear function over the entire KI concentration range used in the experiments (Figure 11). The results showed that the iodide quenching effect was higher for compound P1 than for its complex with ctDNA. The value of K_SV_ for the complex P1 with ctDNA is 27.50·10^3^ M^−1^ and for the sample without the macromolecule it is higher and equal to 31.02·10^3^ M^−1^ (Table 6). However, the difference is still very small. In the case of compound P2, K_SV_ constants without ctDNA (21.46·10^3^ M^−1^) and with ctDNA (21.84·10^3^ M^−1^) are comparable (Table 7). Electrostatic bonds on the surface of the nucleic acid helix and grooves provide significantly less protection for the fluorophore because they are exposed to the external environment and are more readily accessible to iodide ions than intercalators, even in the presence of DNA [54,55]. The relatively small differences observed between the free and DNA-bound peptides suggest that the fluorophores remain largely accessible to the quencher even after complex formation. These results do not confirm a significant degree of protection of the fluorophores by the DNA double helix, as might be expected in the case of strong intercalation-type binding. Instead, the fluorescence quenching data indicate a dominant interaction mechanism other than intercalation and may suggest binding within the helix groove or another mode of binding accessible from the outside. Nevertheless, Stern–Volmer analysis alone does not allow for an unequivocal determination of the DNA binding mode.

**Figure 12:**
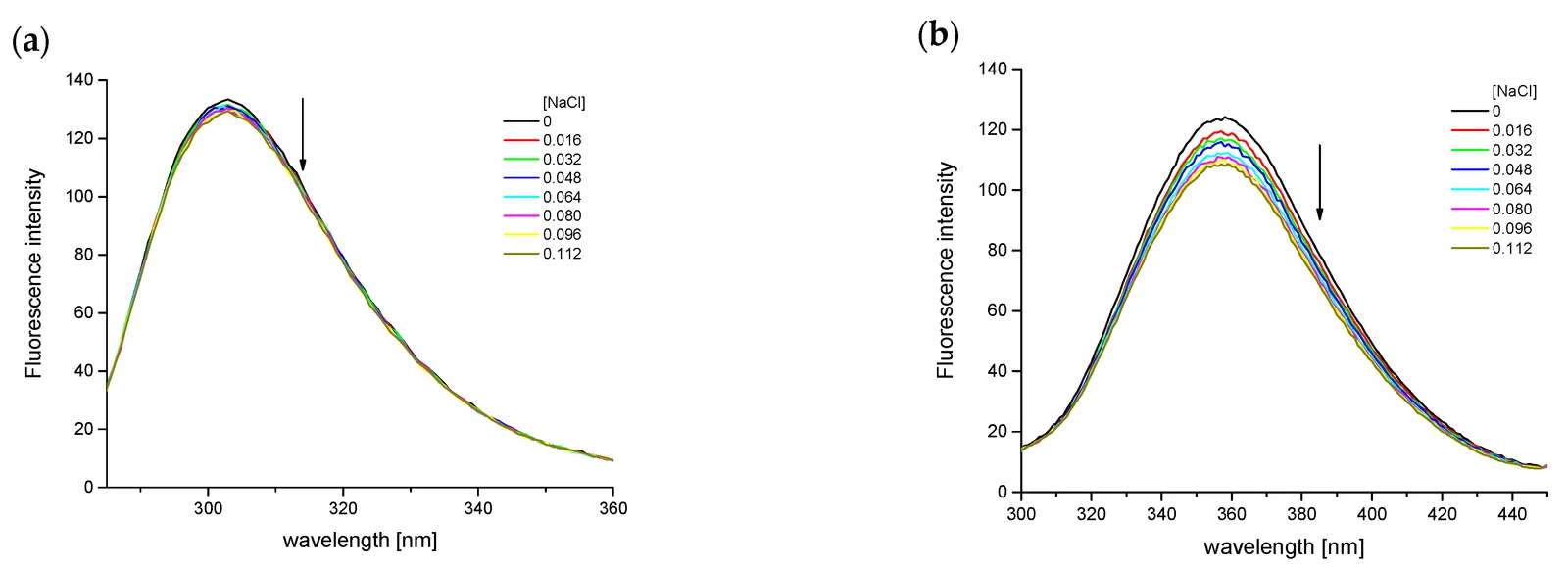
(**a**) Fluorescence spectra of the P1 complex with ctDNA at various NaCl concentrations. (**b**) Fluorescence spectra of the P2 complex with ctDNA at various NaCl concentrations. The arrow indicates the direction of the change in fluorescence intensity observed with increasing concentration NaCl.

The interaction of compounds with the structure of DNA can be assessed by monitoring changes in fluorescence intensity in response to forced changes in ionic strength. This approach enables the evaluation of the binding mechanism and nature of DNA ligand interactions. The complexes of compounds P1 and P2 are not very sensitive to increasing ionic strength, as the intensity of the fluorescence signals changes only slightly (Figure 12). The complexes are stable under salinity conditions close to physiological concentrations. Changes in the emission profile of the ligand/ctDNA complex with increasing ionic strength may suggest electrostatic interactions between the ligand and the biomolecule. Comparing both compounds, it can be concluded that the slightly larger response observed for P2 upon increasing ionic strength may suggest a greater contribution of this type of interaction compared with P1 (Figure 12).

**Figure 13:**
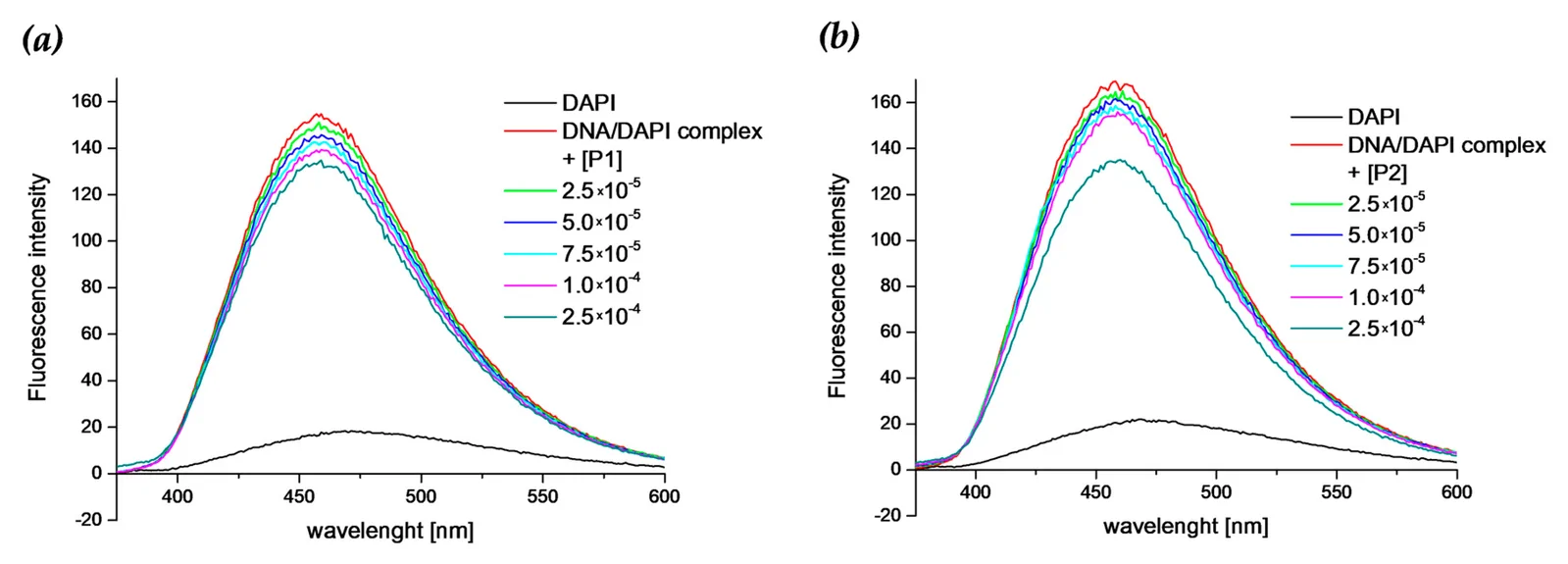
The fluorescence spectra of the titration of ctDNA (4.0·10^−5^ M) complex with DAPI (2.0·10^−6^ M) by: (**a**) P1, and (**b**) P2.

A correlative binding study was carried out using DNA markers, each characterized by a well-defined binding mechanism. Changes in the fluorescence of a DNA-bound marker upon addition of a ligand may indicate perturbation of the probe environment or possible competition for an overlapping binding region [56,57,58]. The DAPI molecule has been observed to interact with the DNA molecule [59,60,61]. The mechanism of DAPI binding to DNA has been comprehensively delineated in extant literature. It has been demonstrated that DAPI exhibits a preference for binding to the minor groove of regions that are rich in A–T pairs [59]. This binding is accompanied by a substantial increase in fluorescence intensity and a change in the spectroscopic properties of the DAPI–DNA complex. DAPI is a fluorescent dye molecule that is of limited utility due to its low intensity. However, its emission is significantly increased after binding within the DNA base pairs [62]. The fluorescence measurements were carried out by titration of marker/ctDNA complexes with a small amount of the studied compounds. Subsequent to the addition, a decline in peak height was detected. The decrease in DAPI–ctDNA fluorescence was calculated by Equation (3). The decreasing fluorescence intensity is presented in Figure 13a,b. The percentage of decrease in DAPI–ctDNA fluorescence was found to be 13.23% for the studied compound P1 and 20.71% for P2. The values obtained are not big. Therefore, they may merely indicate the possibility of competition or overlap with the DAPI binding region. However, the decrease in DAPI–ctDNA fluorescence does not independently establish DAPI displacement or minor-groove occupation by P1 or P2.

**Figure 14:**
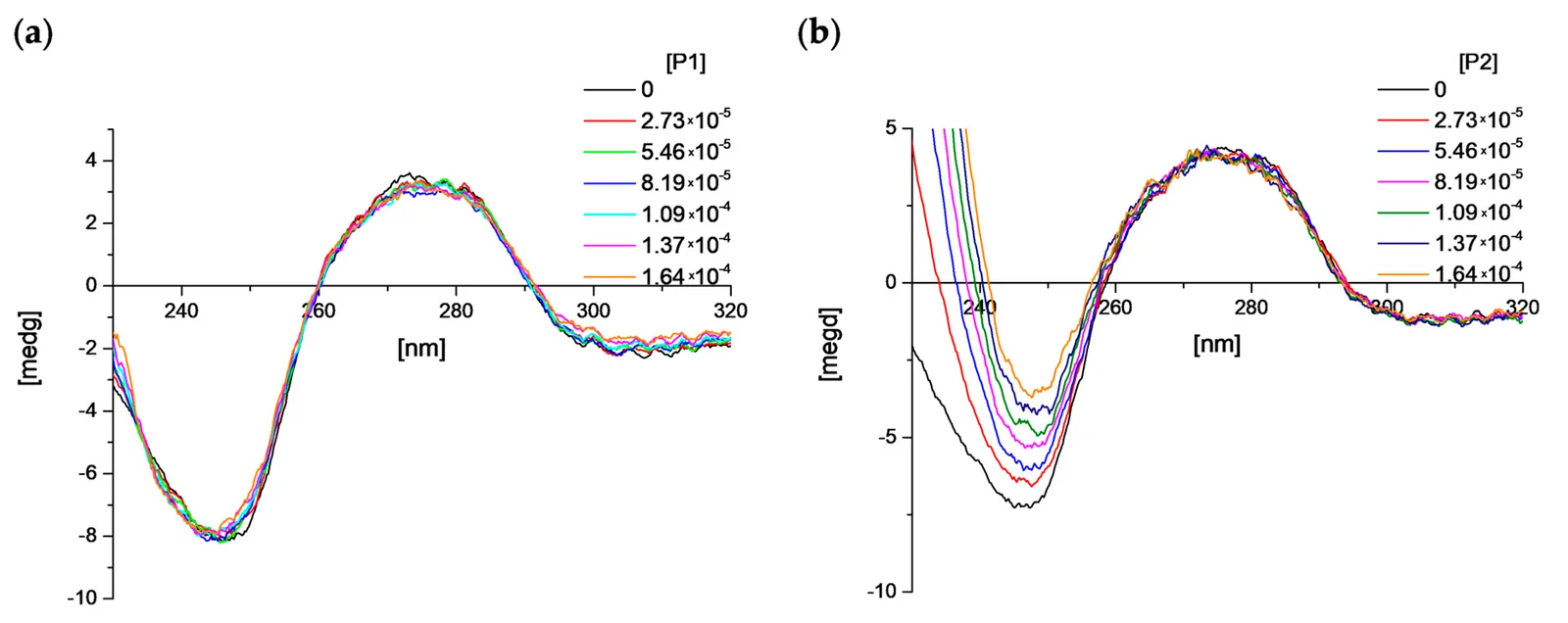
The circular dichroism spectra of ctDNA with the addition of various quantities of compound solutions: (**a**) P1, (**b**) P2.

Analysis of the interaction of the tested peptides with the deoxyribonucleic acid molecule was also performed using CD spectroscopy. The obtained spectra are presented in Figure 14. Each reported spectrum represents the average of three consecutive scans recorded under identical conditions to improve the signal-to-noise ratio and assess short-term spectral reproducibility. This method allows for determining the effect of the tested compounds on the spatial structure of the nucleic acid molecule. This makes it easier to determine the mechanism of interaction in the tested systems.

The obtained spectra are typical for the B-form of ctDNA [63]. Each of the recorded spectra displays two bands: negative at approximately 245 nm, attributable to the right-handed helix, and positive at 275 nm, which is ascribed to base pair stacking [64]. According to earlier research, the nature of interactions within the studied systems can be inferred by analyzing variations in the CD spectra that occur during the binding of a ligand to the ctDNA molecule. Intercalative binding typically leads to pronounced spectral changes, including shifts in band positions and significant variations in intensity. In contrast, interactions involving minor-groove binding or electrostatic forces tend to produce only minimal spectral modifications [65,66]. In the case of the P1/ctDNA system, no major changes in the spectra are observed with increasing concentration of the tested peptide (Figure 14a). The position of the two bands and their intensity remain constant. The results obtained in this study corroborate the conclusions that were drawn from the previously described experiments. A mechanism of interaction other than intercalation is likely here. The other case is slightly different for the P2 peptide (Figure 14b). The CD spectra obtained demonstrate an absence of substantial alterations in the band around 275 nm. However, a marked change in the intensity of the negative band at 245 nm is observed. Consequently, it can be deduced that the P2 compound exerts a more pronounced effect on the spatial structure of the ctDNA molecule. In this instance, a slight disturbance to the right-handed helix is observed. A comparable situation was observed in the case of the interaction of coptisine with a deoxyribonucleic acid molecule [67]. Nevertheless, considering that the second band remains largely unchanged and no new bands appear, the results do not support a predominantly intercalative mode of P2. As for P1, the spectral changes are consistent with non-intercalative. It can therefore be assumed that a minor-groove binding mechanism is likely for both peptides; however, the CD method does not allow for its unequivocal confirmation.

**Figure 15:**
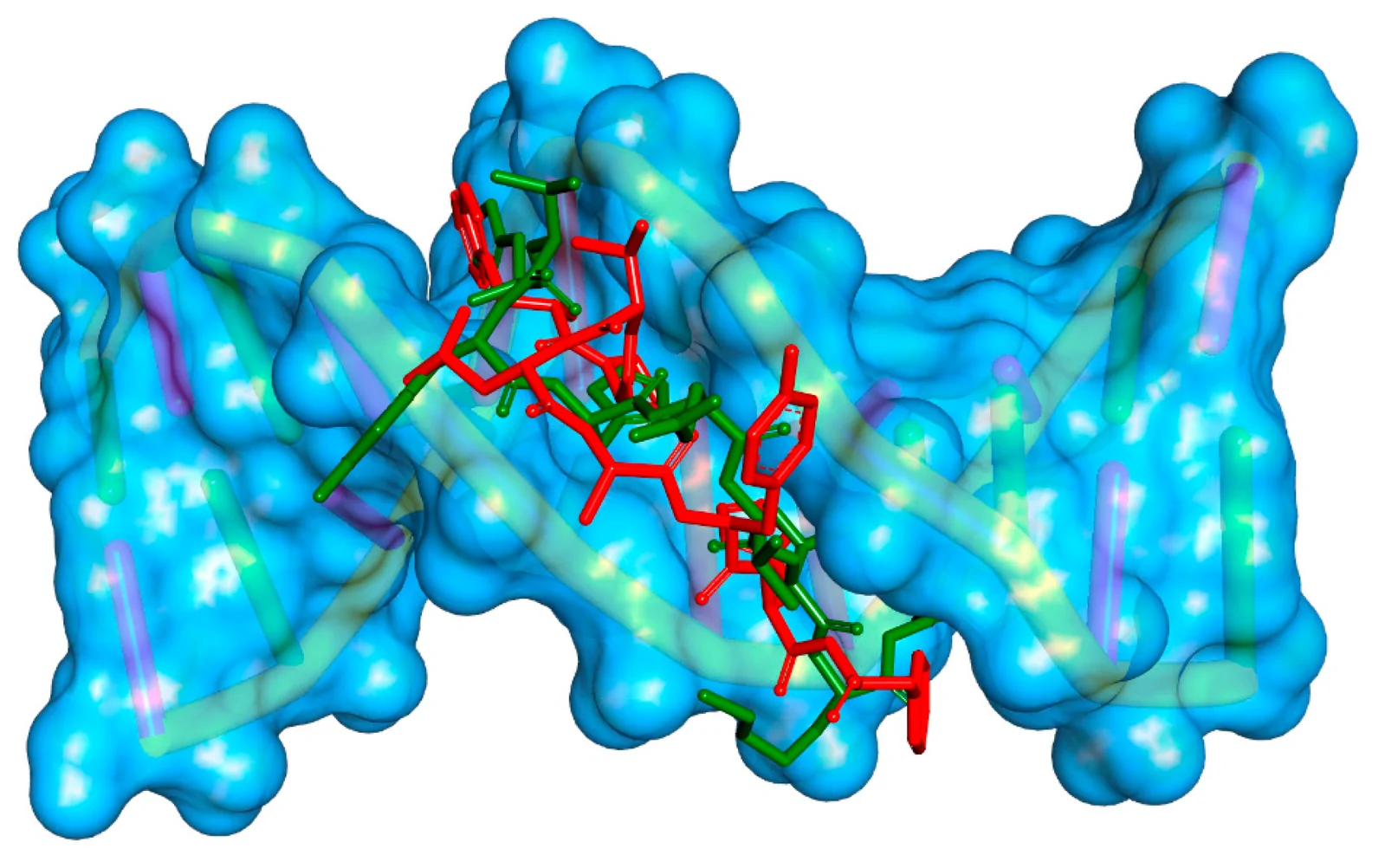
Binding poses of peptide P1 (green) and P2 (red) within the minor groove of DNA.

To further explore the possible interaction modes of compounds P1 and P2 with DNA, molecular docking was performed using the entire DNA structure. The predicted docking poses of both peptides were located within the minor groove of DNA (Figure 15). In the DNA-P1 complex, hydrogen bonds are formed between K8 and Thymine DT19, H9 and Guanine DG19, H9 and Thymine DT20, T2 and Cytosine DC9, as well as T2 and Guanine DG10. There are also π-anion interactions involving Y3-DT20 and π-π, T-shaped, between H1 and DC11. For the DNA-P2 complex, the following interactions are observed: hydrogen bonding (W1-DT7 and H7-DC9), π-anion involving F8-DC11, and π-alkyl interaction between P2-DT8, DA18 (Figures S9 and S10).

Although the relatively short sequences of P1 and P2 do not inherently preclude access to intracellular or nuclear compartments, the effective delivery of free peptides may be limited by proteolytic instability, insufficient membrane permeability, and endosomal sequestration. Importantly, these barriers can potentially be addressed using contemporary delivery strategies, including encapsulation in PEGylated or pH-sensitive liposomes, ionizable lipid or polymeric nanoparticles, polymeric micelles, as well as conjugation with cell-penetrating peptides or nuclear localization sequences. Such approaches have been shown to enhance peptide stability, cellular uptake, endosomal escape and, in appropriately designed systems, nuclear accumulation [68,69]. Future studies involving fluorescently labeled P1 and P2, flow cytometry, confocal microscopy, and subcellular fractionation, together with comparisons of free and carrier-formulated peptides, could therefore clarify the biological relevance of the ctDNA interactions observed in vitro.

The ctDNA experiments further demonstrate that both peptides can interact with duplex DNA under cell-free conditions, with the results being consistent with a non-intercalative association. As these experiments were performed in a cell-free model, further studies in cellular systems will be required to assess the biological relevance of these interactions, including peptide stability and cellular internalization.

The obtained spectroscopic and theoretical results indicate that the probable mechanism of interaction between the studied Babylon snail-derived peptides and DNA is non-intercalative. Other types of interactions are possible, among which binding within the minor groove of the double helix is likely. From a pharmacological perspective, such a mechanism could potentially be beneficial, as it does not cause extensive disruption to the spatial structure of the DNA molecule—unlike intercalation. Minor-groove binders constitute a large group of compounds with anticancer or antibacterial activity. They can modulate biological processes by selectively interacting with DNA regions involved in the activity of transcription factors, polymerases, and other regulatory proteins. Consequently, they influence gene expression, DNA replication, and DNA repair pathways, while maintaining a reversible mode of binding to the macromolecule [70]. Thus, the peptides under study represent attractive scaffolds for the development of biologically active compounds. However, this assumption naturally requires further confirmation through in vitro and in vivo studies.

Summarizing the experimental results described in this work, it should be noted that the peptide concentrations used in these in vitro studies were selected primarily to ensure adequate spectroscopic sensitivity and the reliable determination of interaction parameters. Consequently, they do not correspond to concentrations achieved in vivo. Therefore, the observed peptide–biomolecule interactions should not be directly extrapolated to issues regarding peptide distribution, bioavailability, or pharmacological activity, as these depend on additional factors such as absorption, proteolytic stability, plasma protein binding, tissue distribution, and clearance.

## 4. Conclusions

In this study we described the interaction of Babylon snail-derived peptides P1 and P2 with biopolymers such as bloodstream proteins and ctDNA. A thorough examination of the findings indicates interaction between compounds P1 and P2 and plasma proteins. The apparent association constants were on the order of 10^3^ M^−1^ under the applied experimental conditions. The complexation process is thermodynamically favorable under the experimental conditions and induces only minor changes in the global conformation of the studied proteins. Molecular modeling suggests the possibility of the studied peptides binding in both albumin pockets: Sudlow I and II. Results also indicate that hydrogen bonding could play a dominant role in complex stabilization, while hydrophobic interactions provide additional enhancement of the stability of complexes. Meanwhile, experimental data showed that both P1 and P2 caused a decrease in the fluorescence intensity of the QR–AAG system, indicating partial displacement of the probe from the binding site within the protein. This observation suggests that the peptides interact with the same binding region as QR or with a region that partially overlaps with it. Furthermore, molecular docking analysis predicted binding modes characterized by potential hydrogen bonds and hydrophobic interactions within the identified binding pocket. Molecular docking provides a static prediction of plausible binding modes. Molecular dynamics simulations would be required to further assess the persistence and conformational stability of the predicted interactions.

Furthermore, the compounds under investigation interact with ctDNA molecules. The spectroscopic results obtained rule out an intercalation model of interaction, thereby suggesting a different mode of binding for the peptides to the DNA molecule. An interaction mechanism involving the minor groove of the double helix is likely. Molecular modeling also predicts this mode of interaction as possible. It can therefore be stated that the combined spectroscopic and docking results are consistent with a predominantly non-intercalative, possibly minor-groove-associated binding mode.

## Figures and Tables

**Table 1:** The binding parameters for the interactions of P1 and P2 with proteins HSA, AAG, IgG, and LYS were obtained using UV-Vis spectral analysis.

Compound	% Chromism	K_a_·10^3^ [dm^3^mol^−1^]	ΔG·10^3^ [Jmol^−1^]
**HSA**			
**P1**	10.21 ± 0.08	1.41 ± 0.08	−17.96 ± 0.14
**P2**	26.73 ± 0.08	1.55 ± 0.07	−18.19 ± 0.10
**AAG**			
**P1**	38.42 ± 0.15	2.52 ± 0.30	−19.40 ± 0.30
**P2**	59.23 ± 0.15	2.27 ± 0.50	−19.15 ± 0.55
**LYS**			
**P1**	35.48 ± 0.32	3.85 ± 0.15	−20.45 ± 0.10
**P2**	61.45 ± 0.44	3.72 ± 0.04	−20.37 ± 0.08
**IgG**			
**P1**	2.87 ± 0.04	-	-
**P2**	6.50 ± 0.04	-	-

**Table 2:** The changes in the secondary structure of HSA induced by increasing the concentration of P1 or P2. A quantitative analysis was conducted in the CD Multivariate SSE program using the PCR method, utilizing averaged spectra of HSA (1∙10^−6^ M) with five accumulations.

Peptide Concentration [mol/dm^3^]	% α-Helix	% β-Sheet	% β-Turn	% Other	RMSD
**P1**					
0	62.9	3.9	9.7	23.5	0.0020
0.8∙10^−6^	62.5	4.6	9.7	23.2	0.0022
2.0∙10^−6^	62.2	4.3	9.8	23.7	0.0024
**P2**					
0	64.1	3.3	9.6	23.0	0.0021
0.8∙10^−6^	63.1	3.8	9.7	23.4	0.0020
2.0∙10^−6^	62.9	4.0	9.7	23.4	0.0021

**Table 3:** The changes in the secondary structure of AAG induced by increasing the concentration of P1 or P2. A quantitative analysis was conducted in the CD Multivariate SSE program using the PCR method, utilizing averaged spectra of AAG (1∙10^−6^ M) with five accumulations.

Peptide Concentration [mol/dm^3^]	% α-Helix	% β-Sheet	% β-Turn	% Other	RMSD
**P1**					
0	25.5	34.1	10.4	30.0	0.0025
0.8∙10^−6^	25.8	34.1	10.3	29.8	0.0023
2.0∙10^−6^	26.7	32.9	10.5	29.9	0.0021
**P2**					
0	26.8	33.6	10.4	29.2	0.0024
0.8∙10^−6^	27.0	33.7	10.3	29.0	0.0020
2.0∙10^−6^	27.2	34.2	10.1	28.5	0.0023

**Table 4:** The changes in the secondary structure of LYS induced by increasing the concentration of P1 or P2. A quantitative analysis was conducted in the CD Multivariate SSE program using the PCR method, utilizing averaged spectra of LYS (1∙10^−6^ M) with five accumulations.

Peptide Concentration [mol/dm^3^]	% α-Helix	% β-Sheet	% β-Turn	% Other	RMSD
**P1**					
0	32.1	21.0	12.4	34.5	0.0024
0.8∙10^−6^	31.3	21.0	12.7	35.0	0.0023
2.0∙10^−6^	30.4	20.7	12.7	36.2	0.0025
**P2**					
0	32.3	20.7	12.4	34.6	0.0023
0.8∙10^−6^	31.2	20.9	12.5	35.4	0.0025
2.0∙10^−6^	30.2	21.3	12.7	35.8	0.0025

**Table 5:** The changes in the secondary structure of IgG induced by increasing the concentration of P1 or P2. A quantitative analysis was conducted in CD Multivariate SSE program using the PCR method, utilizing averaged spectra of IgG (1∙10^−6^ M) with five accumulations.

Peptide Concentration [mol/dm^3^]	% α-Helix	% β-Sheet	% β-Turn	% Other	RMSD
**P1**					
0	6.4	39.7	13.3	40.5	0.0022
0.8∙10^−6^	6.6	40.4	13.2	39.7	0.0024
2.0∙10^−6^	6.7	40.0	13.2	40.0	0.0025
**P2**					
0	6.6	40.0	13.3	40.1	0.0021
0.8∙10^−6^	6.6	40.2	13.2	39.9	0.0023
2.0∙10^−6^	6.4	40.5	13.2	39.8	0.0023

**Table 6:** The percentage of fluorescence exchanged dye in the proteins HSA and AAG by the studied compounds P1 and P2.

Marker/Protein Complex	%E	
	P1	P2
**dGly/HSA**	28.6	37.2
**dPhe/HSA**	28.9	37.6
**QR/AAG**	53.0	52.2

**Table 7:** The binding parameters for ctDNA interaction with P1 and P2: the percentage of chromicity effect, the binding constant (K_a_), and the standard Gibbs free energy (ΔG) calculated from UV-Vis spectra analysis, and Stern–Volmer (K_SV_) constants calculated from fluorescence spectra analysis.

Compound	% Chromism	K_a_·10^3^ [dm^3^mol^−1^]	ΔG·10^3^ [Jmol^−1^]	K_SV_·10^3^ [dm^3^mol^−1^]	
				with ctDNA	Without ctDNA
**P1**	71.00 ± 0.52	0.75 ± 0.08	−16.43 ± 0.16	27.50 ± 1.10	31.02 ± 1.41
**P2**	43.94 ± 0.35	1.78 ± 0.21	−18.55 ± 0.24	21.84 ± 0.70	21.46 ± 0.81

## Data Availability

Data is contained within the article or supplementary material.

## Supplementary Materials

The following supporting information can be downloaded at https://www.mdpi.com/article/10.3390/pharmaceutics18101311/s1, Figure S1. 2D plot of interactions of P1 with HSA in site I; Figure S2. 2D plot of interactions of P2 with HSA in site I; Figure S3. 2D plot of interactions of P1 with HSA in site II; Figure S4. 2D plot of interactions of P2 with HSA in site II; Figure S5. 2D plot of interactions of P1 with AAG; Figure S6. 2D plot of interactions of P2 with AAG; Figure S7. 2D plot of interactions of P1 with LYS; Figure S8. 2D plot of interactions of P2 with AAG; Figure S9. 2D plot of interactions of P1 with DNA; Figure S10. 2D plot of interactions of P2 with DNA.

## Abbreviations

The following abbreviations are used in this manuscript:

AAG	α1-acid glycoprotein acid
ctDNA	calf thymus deoxyribonucleic acid
DAPI	4′,6-diamidine-2′-phenylindole dihydrochloride
DGly	dansylated-L-glycine
DPhe	dansylated-L-phenylalanine
HSA	human serum albumin
LYS	human lysozyme
IgG	human immunoglobulin G
QR	quinaldine red
RMSD	Root Mean Square Deviation
	single-letter of amino acid abbreviations were used
