# Supplementary material for: Molecular Interactions of Bioactive Peptides with Proteins and DNA
Source: Pharmaceutics. 2026 Oct 15;18(10):1311. doi: 10.3390/pharmaceutics18101311 (PMC13708310; doi:10.3390/pharmaceutics18101311)
Supplement: Supplementary file 1 [file pharmaceutics-18-01311-s001.zip › pharmaceutics-4583154-supplementary.pdf]

Molecular interactions of bioactive peptides with proteins and DNA

Aleksandra Kotynia[1]*, Edward Krzyzak[1], Żaneta Czyżnikowska[1] and Aleksandra Marciniak[1]

[1] Department of Basic Chemical Sciences, Faculty of Pharmacy, Wroclaw Medical University, Borowska 211a, 50-556, Wrocław, Poland; aleksandra.kotynia@umw.edu.pl (A.K.); edward.krzyzak@umw.edu.pl (E.K.); zaneta.czyznikowska@umw.edu.pl (Ż.C.); aleksandra.marciniak@umw.edu.pl (A.M.)

* Correspondence: aleksandra.kotynia@umw.edu.pl

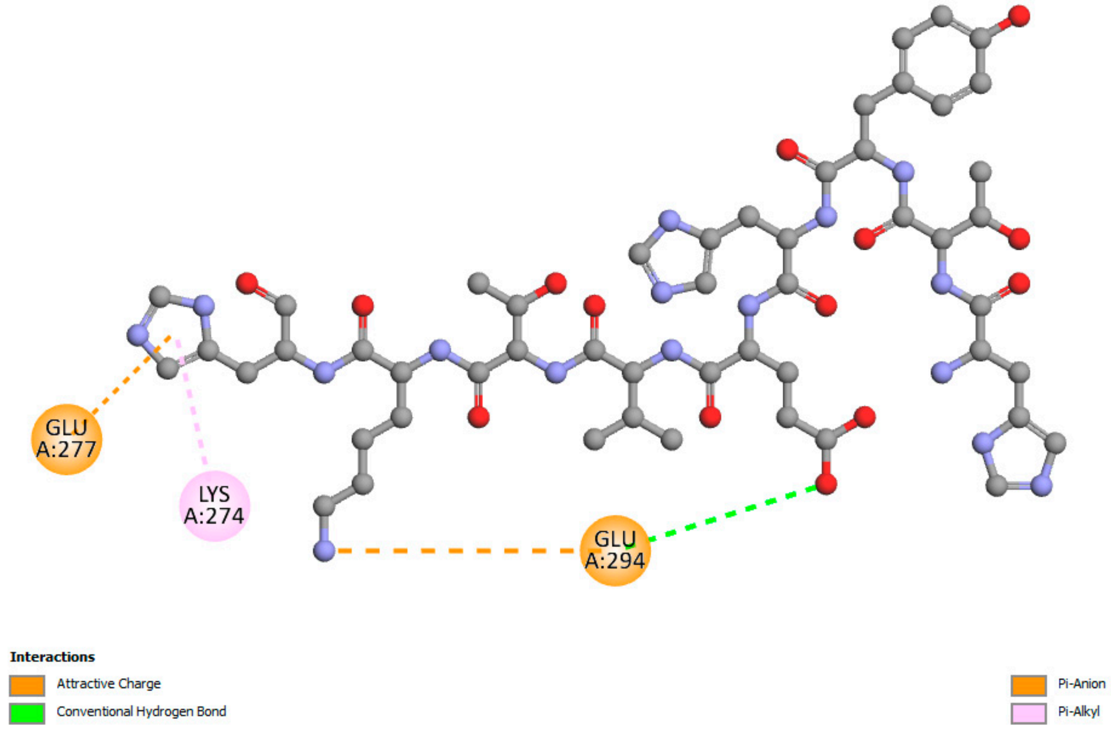

Figure S1. 2D plot of interactions of P1 with HSA in site I.

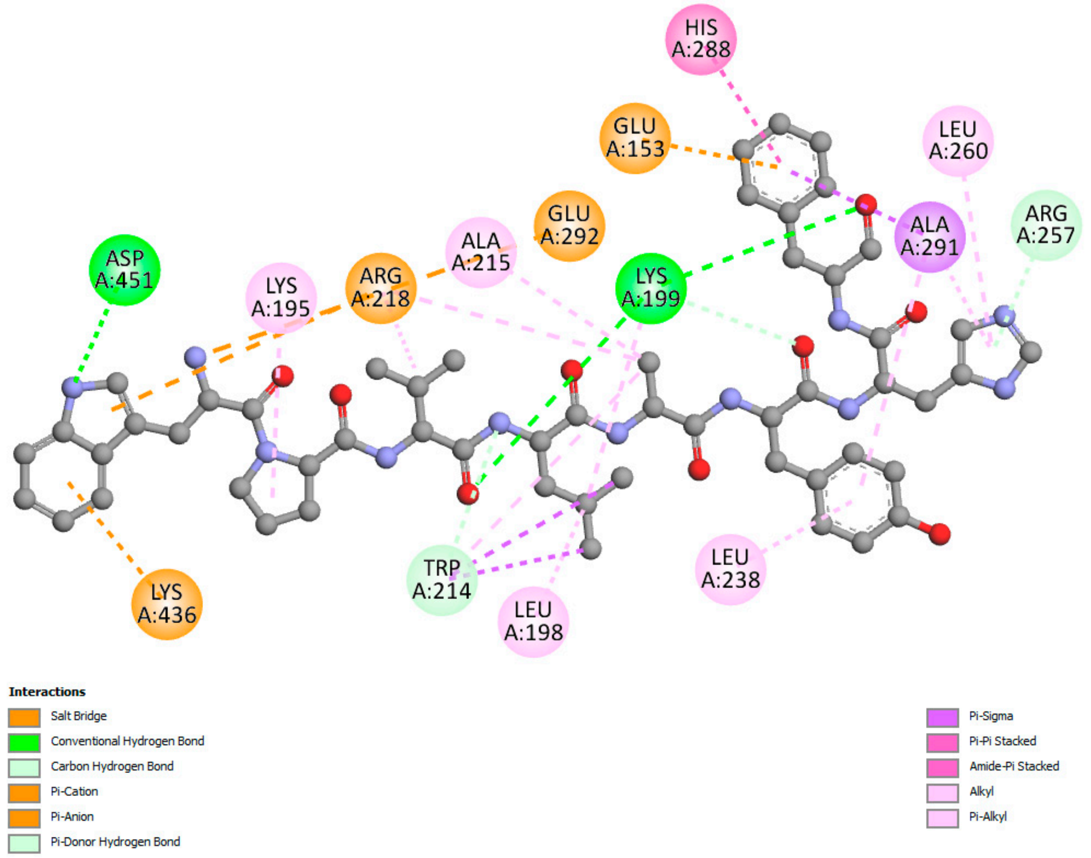

Figure S2. 2D plot of interactions of P2 with HSA in site I.

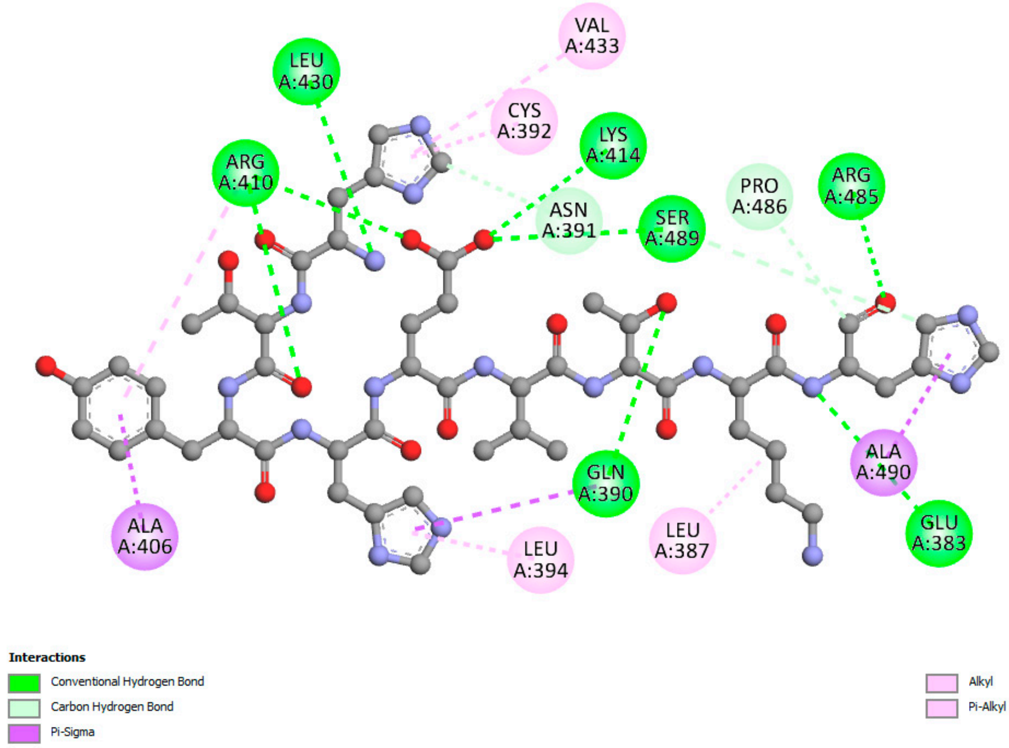

Figure S3. 2D plot of interactions of P1 with HSA in site II.

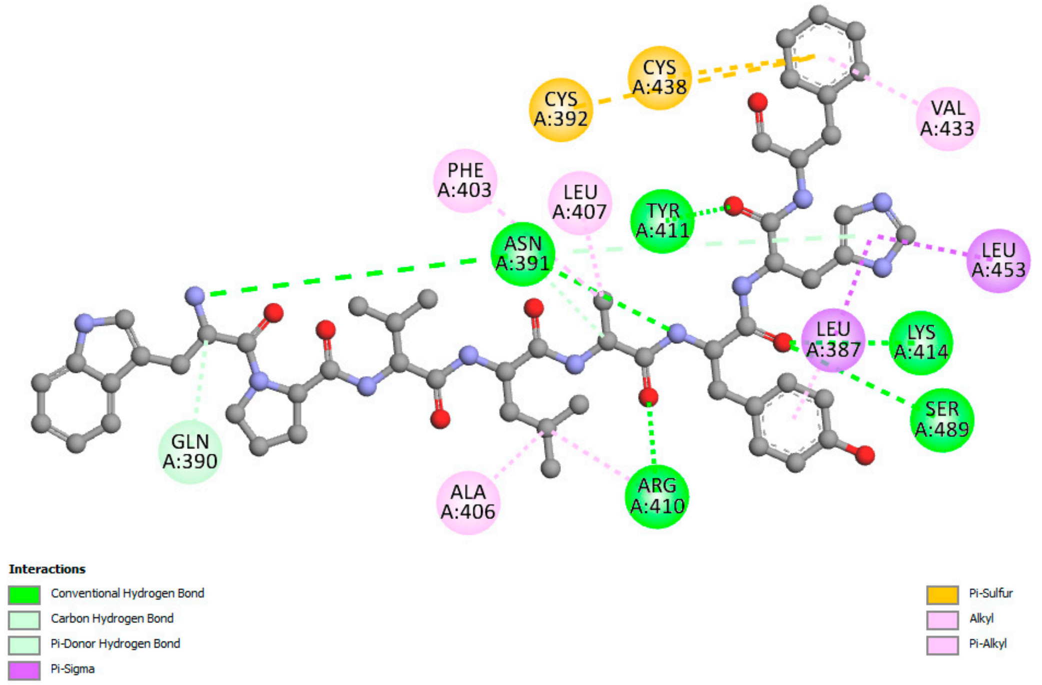

Figure S4. 2D plot of interactions of P2 with HSA in site II.

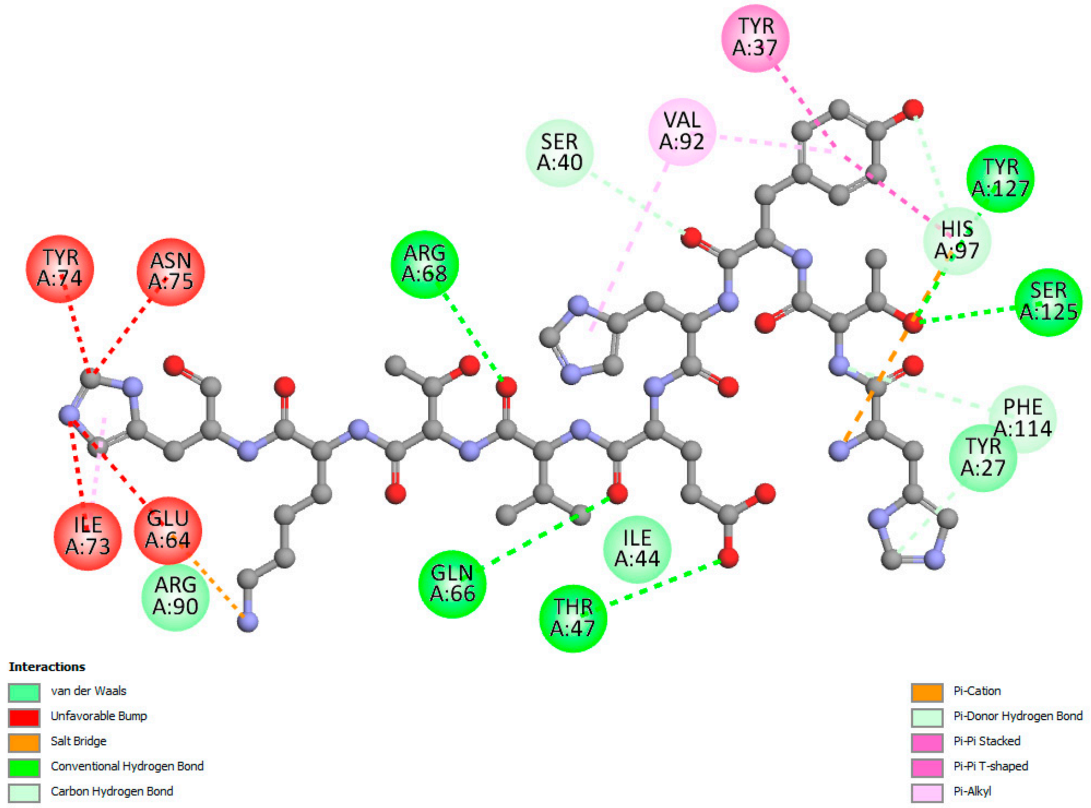

Figure S5. 2D plot of interactions of P1 with AAG.

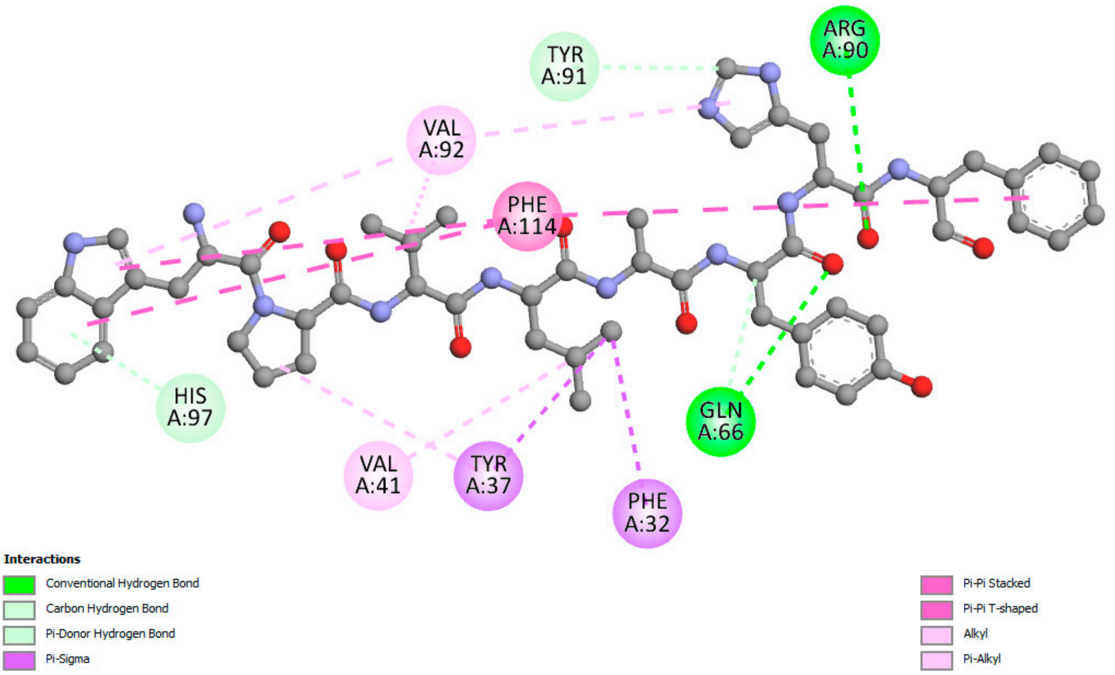

Figure S6. 2D plot of interactions of P2 with AAG.

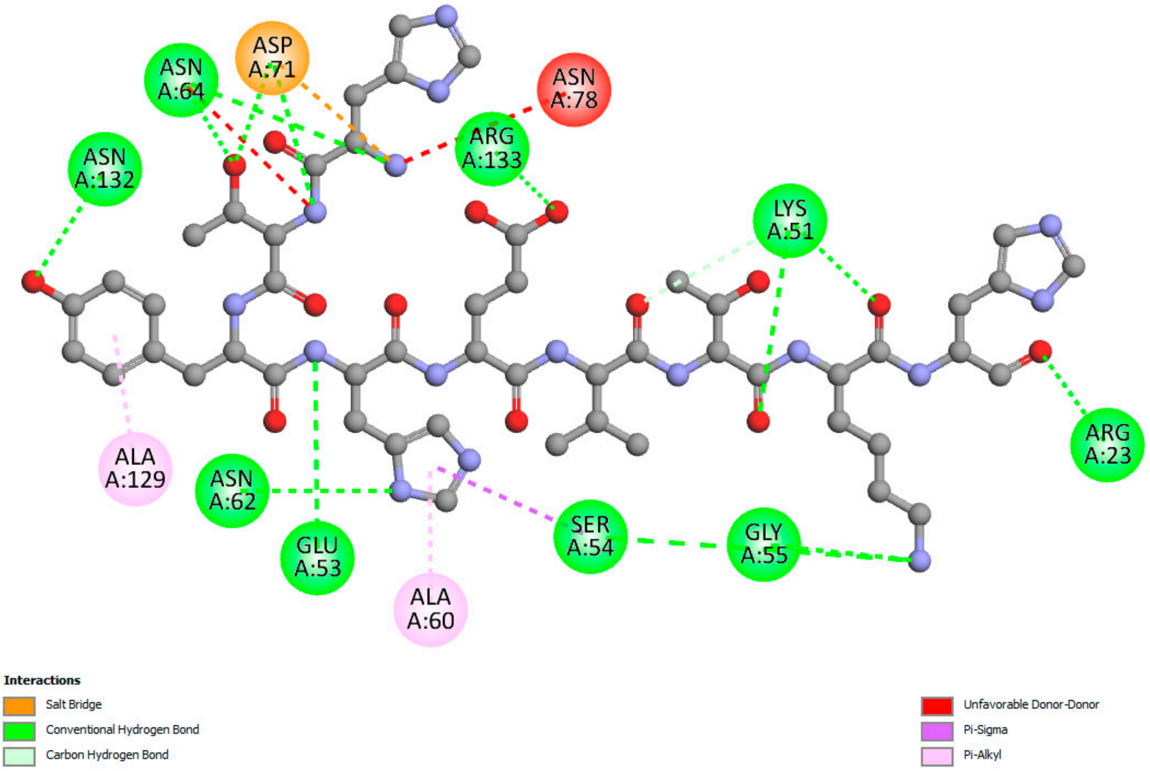

Figure S7. 2D plot of interactions of P1 with LYS.

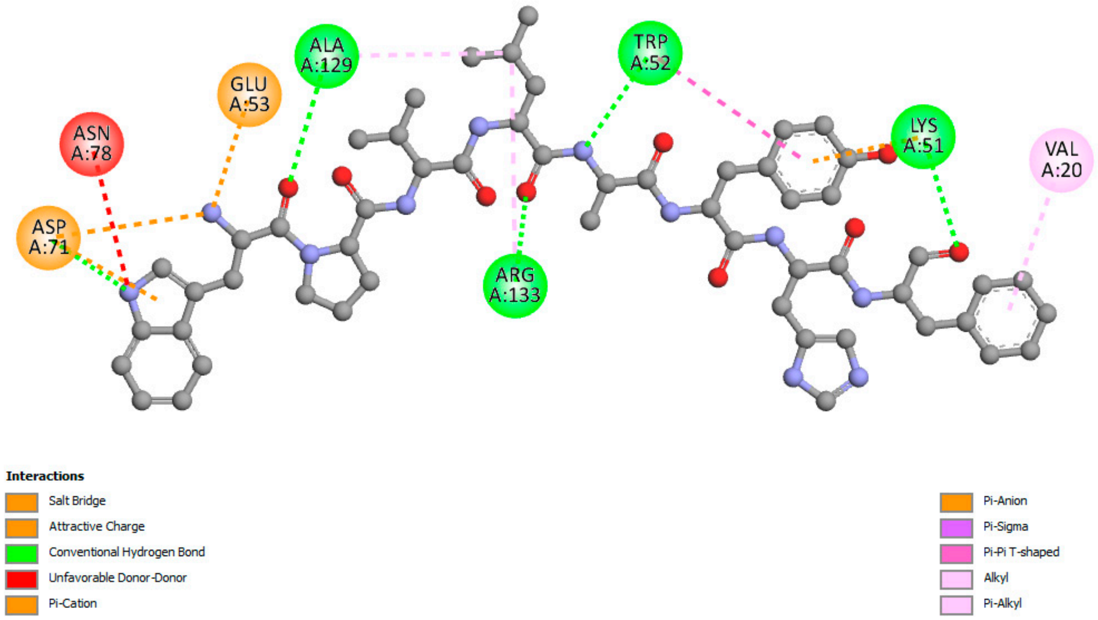

Figure S8. 2D plot of interactions of P2 with AAG.

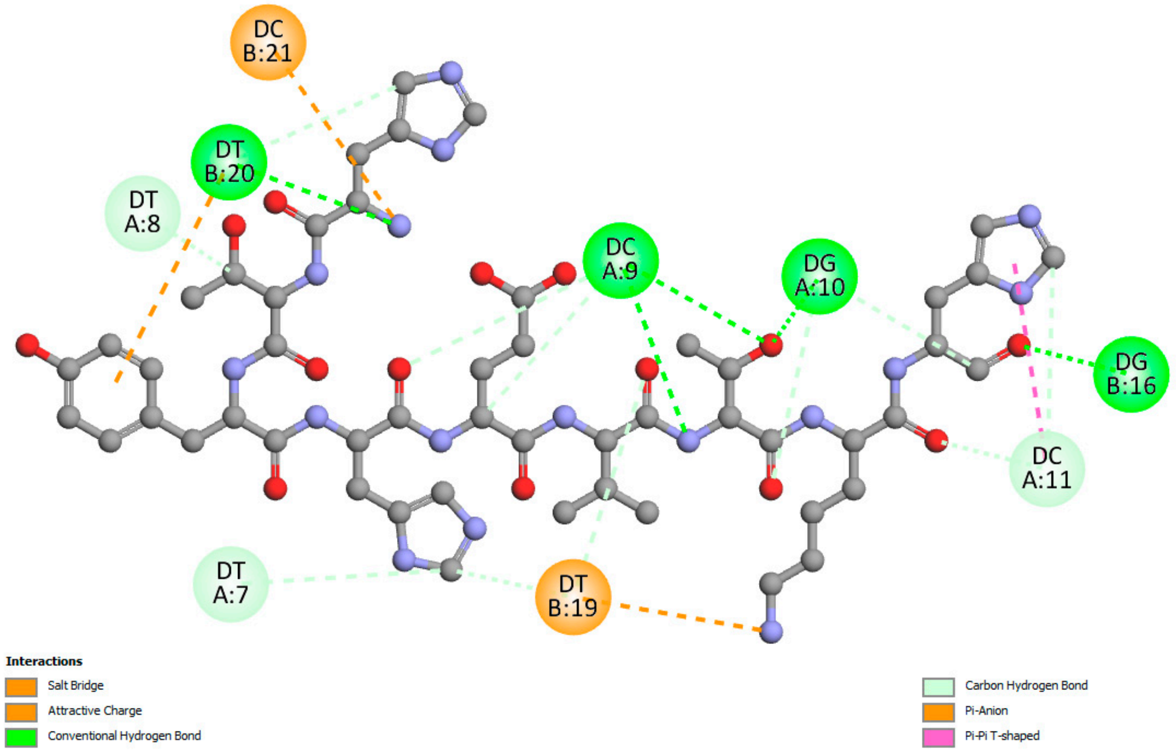

Figure S9. 2D plot of interactions of P1 with DNA.

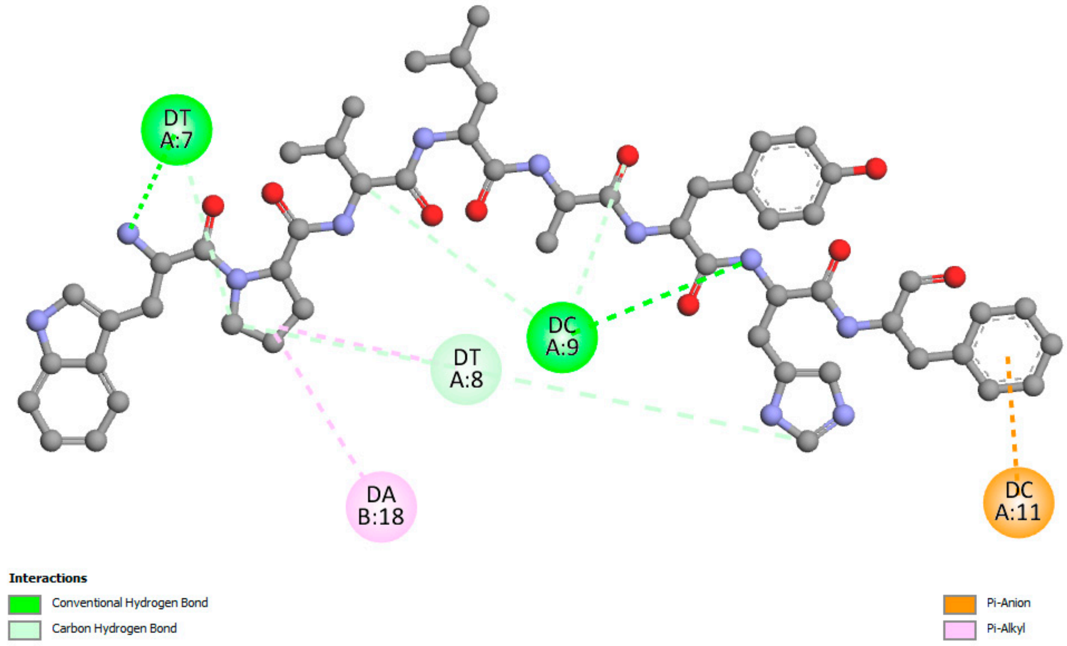

Figure S10. 2D plot of interactions of P2 with DNA.
